# Early embryogenesis in CHDFIDD mouse model reveals facial clefts and altered cranial neurogenesis

**DOI:** 10.1242/dmm.050261

**Published:** 2024-06-20

**Authors:** Marek Hampl, Nela Jandová, Denisa Lusková, Monika Nováková, Tereza Szotkowská, Štěpán Čada, Jan Procházka, Jiri Kohoutek, Marcela Buchtová

**Affiliations:** ^1^ Laboratory of Molecular Morphogenesis, Institute of Animal Physiology and Genetics, Czech Academy of Sciences, 60200 Brno, Czech Republic; ^2^ Department of Experimental Biology, Faculty of Science, Masaryk University, 60200 Brno, Czech Republic; ^3^ Department of Chemistry and Toxicology, Veterinary Research Institute, 62100 Brno, Czech Republic; ^4^ Laboratory of Transgenic Models of Diseases, Institute of Molecular Genetics, Czech Academy of Sciences, 14220 Prague, Czech Republic; ^5^ Czech Centre for Phenogenomics, Institute of Molecular Genetics, Czech Academy of Sciences, 14220 Prague, Czech Republic

**Keywords:** Craniofacial development, Orofacial clefts, Axons, Neurite outgrowth, CDK13, Trigeminal ganglion

## Abstract

CDK13-related disorder, also known as congenital heart defects, dysmorphic facial features and intellectual developmental disorder (CHDFIDD) is associated with mutations in the *CDK13* gene encoding transcription-regulating cyclin-dependent kinase 13 (CDK13). Here, we focused on the development of craniofacial structures and analyzed early embryonic stages in CHDFIDD mouse models, with one model comprising a hypomorphic mutation in *Cdk13* and exhibiting cleft lip/palate, and another model comprising knockout of *Cdk13*, featuring a stronger phenotype including midfacial cleft. *Cdk13* was found to be physiologically expressed at high levels in the mouse embryonic craniofacial structures, namely in the forebrain, nasal epithelium and maxillary mesenchyme. We also uncovered that *Cdk13* deficiency leads to development of hypoplastic branches of the trigeminal nerve including the maxillary branch. Additionally, we detected significant changes in the expression levels of genes involved in neurogenesis (*Ache*, *Dcx*, *Mef2c*, *Neurog1*, *Ntn1, Pou4f1*) within the developing palatal shelves. These results, together with changes in the expression pattern of other key face-specific genes (*Fgf8, Foxd1*, *Msx1*, *Meis2 and Shh*) at early stages in *Cdk13* mutant embryos, demonstrate a key role of CDK13 in the regulation of craniofacial morphogenesis.

## Teaser

**Summary:** CDK13 plays a role in the regulation of facial morphogenesis as well as the growth of cranial peripheral nerves.

**Research Simplified**

Congenital heart defects, dysmorphic facial features and intellectual development disorder (CHDFIDD) is a rare genetic disease, mostly reported in children. Reduced levels of CDK13 - a protein essential for the regulation of normal gene expression in human cells - have been implicated in some patients with CHDFIDD.

Understanding how CDK13 deficiency affects early development during pregnancy can help researchers devise therapeutics for CDK13-related CHDFIDD.

Since humans and mice share several developmental and anatomical similarities, the authors firstly used established laboratory mouse models with mutations in the CDK13-coding gene to mimic CDK13 deficiencies seen in humans with CHDFIDD. Mouse embryos with reduced CDK13 expression displayed structural defects in their heads, mostly in the face, as well as underdeveloped facial nerves. The authors find that these anomalies in CDK13-deficient mice were caused by lowered expression of genes with critical roles in the development of the face and in nerve growth.

This study tracked the expression of CDK13 during different developmental stages of the mouse embryo and confirmed the importance of this protein in craniofacial and neural development. Further research can facilitate the development of potential therapeutics for humans with CDK13-related CHDFIDD.

## INTRODUCTION

CDK13 is one of the transcriptional kinases that regulates transcription via phosphorylation of RNA polymerase II (RNAPII) and controls alternative splicing (Bartkowiak et al., 2010; Blazek et al., 2011). Recently, a few case studies have described a variety of developmental defects in human patients carrying mutated *CDK13* (Hamilton and Suri, 2019). These patients exhibit delayed development, intellectual disorders, heart and kidney defects, and craniofacial anomalies, features that – together – have been recognized as congenital heart defects, dysmorphic facial features and intellectual development disorder (CHDFIDD). In addition to the most common anomalies, patients suffer from brain anomalies, autism, seizures, limb and skeletogenesis anomalies, and other clinical presentations (Hamilton and Suri, 2019). To simulate anomalies presented in patients with *CDK13* mutations, we have developed mouse models that exhibit phenotypes similar to those observed in humans (Nováková et al., 2019). We observed that *Cdk13* deficiency in the mouse models causes embryonic lethality, delayed development, heart and brain abnormalities and a facial phenotype (Nováková et al., 2019).

Here, we screened the gene expression of *Cdk13* across several embryonic craniofacial tissues at different developmental stages, and evaluated the alteration of developmental processes in craniofacial structures in *Cdk13*-hypomorph (*Cdk13^tm1a/tm1a^*) and *Cdk13*-knockout (*Cdk13^tm1d/tm1d^*) mouse embryos, with the aim to uncover the mechanisms behind the observed developmental abnormalities. To determine the modification in molecular regulations caused by *Cdk13* deficiency, we also analyzed the gene expression of patterning proteins involved in craniofacial structure formation.

Moreover, previous *in vitro* approaches have uncovered the contribution of a *Cdk13* mutation to the alteration of neuronal differentiation and neurite outgrowth through regulation of the CDK5 pathway (Chen et al., 2014). These findings are in agreement with observations of neurodevelopmental disorders in human patients carrying mutations of *CDK13* (Trinh et al., 2019). Based on this evidence, we focused here on the analyses of cranial nerve growth and morphology in *Cdk13-*deficient animals, i.e. mice and chicken, that are associated with craniofacial structure formation, including palate morphogenesis. Further, we evaluated possible changes of neurogenesis-specific genes in *Cdk13-*deficient animals and tested the effect of the CDK12/13 inhibitor THZ531 on axon outgrowth from the embryonic trigeminal ganglion.

## RESULTS

### *Cdk13* deficiency causes craniofacial anomalies, including severe facial clefting

**Fig. 1.**
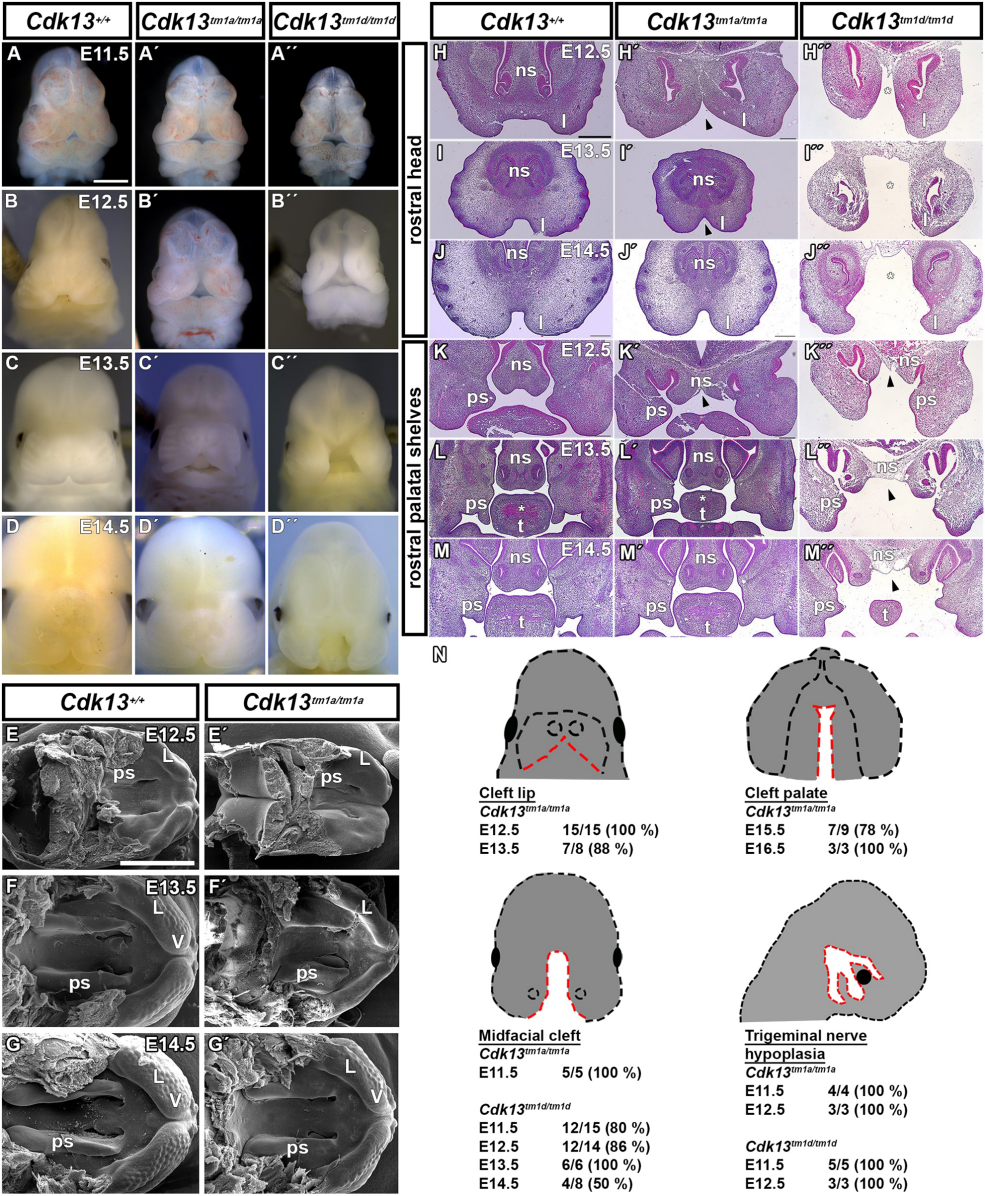
***Cdk13-*deficient embryos display severe craniofacial clefting.** (A-D″) Microscopy images showing external craniofacial phenotypes of WT (*Cdk13^+/+^*), hypomorphic (*Cdk13^tm1a/tm1a^*) and knockout (*Cdk13^tm1d/tm1d^*) mouse embryos at different embryonic stages (E11.5-E14.5). Development of cleft lip typical for hypomorphic *Cdk13*^*tm1a/tm1a*^ embryos is shown in A′-D′. Development of midfacial cleft typical for knockout *Cdk13*^*tm1d/tm1d*^ embryos is shown in A″-D″. (E-G′) Palatal view SEM images of WT and hypomorphic mouse embryos at E12.5, 13.5 and E14.5. Obvious morphological differences of the palatal shelves, as well as the formation of lips and vibrissae can be seen in hypomorphic (E′-G′) compared to WT (E-G) embryos. (H-M″) H&E-stained images of mouse frontal head sections at E12.5 – E14.5. (H–J″) Arrowheads point to the cleft lip in *Cdk13^tm1a/tm1a^* embryos and asterisks highlight the midfacial cleft in KO (*Cdk13^tm1d/tm1d^*) embryos. (K-M″) Arrowheads point to cleft nasal septum in both hypomorph and KO embryos. The palatal shelve development in the rostral region is altered in both genotypes compared to WT embryos. Hypoplastic muscles in the tongue are marked by asterisks. (N) Schematic displaying the frequency of severe craniofacial phenotypes (cleft lip, cleft palate, midfacial cleft, hypoplasia of the trigeminal nerve) within individual genotypes and embryonic developmental stages. Red dashed lines contour anomalous structures. L, lip; ns, nasal septum; ps, palatal shelf; t, tongue; V, vibrissae. Scale bars: 1 mm (A-G′), 500 µm (H-M′′).

In our previous study, we observed craniofacial developmental anomalies caused by *Cdk13* deficiency, which resulted either in a less severe phenotype in hypomorphic embryos [secondary palate dysmorphisms at embryonic day 15.5 (E15.5)] or in a more severe phenotype in knockout embryos (midfacial cleft at E13.5) (Nováková et al., 2019). The morphological difference between these two *Cdk13* genotypes was explained by the residual expression of CDK13 in hypomorphic embryos – probably caused either by insertion of a neomycin selection cassette into the non-coding region, which has been shown to affect gene expression at both RNA and DNA levels, or by a high frequency of post-transcriptional exon shuffling within *Cdk13*, enabling the formation of aberrant functional *Cdk13* transcripts – compared to undetectable expression of CDK13 in knockout embryos (Nováková et al., 2019). Thus, we decided to analyze all the crucial embryonic stages of *Cdk13^tm1a/tm1a^* and *Cdk13^tm1d/tm1d^* mice, all of which exhibited morphological anomalies of craniofacial structures including severe clefting (Fig. 1N).

**Fig. 2.**
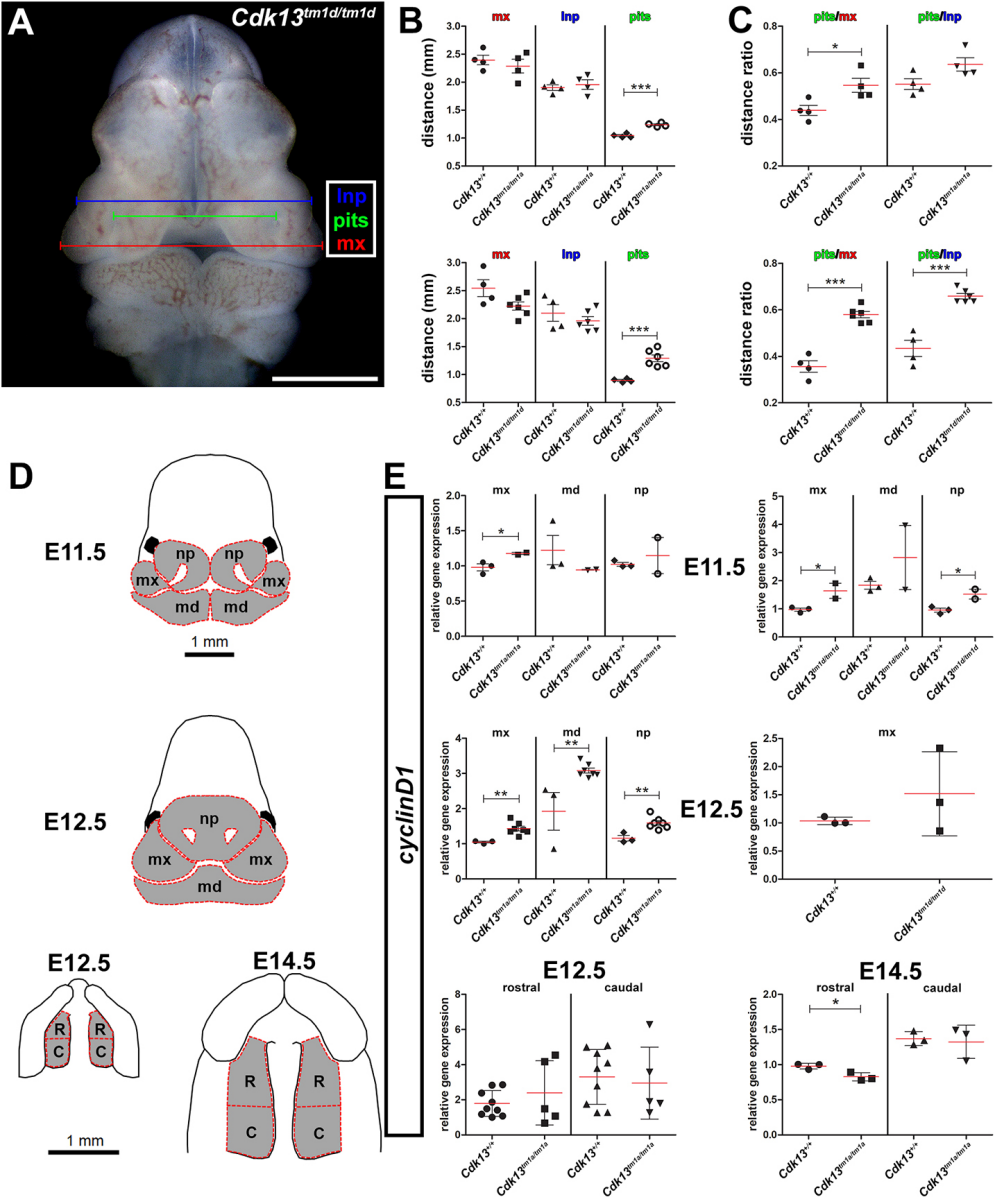
**Changes in facial morphometrics and cell proliferation in *Cdk13* mutant embryos.** (A) Representative microscopy image of a embryonic *Cdk13^tm1d/tm1d^* mouse head outlining the measurements performed at E11.5 in mice as indicated in B-C. Measured were the distances between edges of the lateral nasal prominences (blue), edges of the maxillary prominences (red) and nasal pits (green). (B,C) Individual graphs displaying the distance (B) (in mm) measured in WT, hypomorphic and knockout embryos at E11.5 and the ratio (C) calculated from these distances. (D) Schematics outlining how individual tissues were dissected to quantify *CyclinD1* gene expression in the mx, md and np, and R and C palatal shelves of mice at E11.5 and E12.5. (E) Quantification of the proliferation changes detected in the mx, md and np facial parts, and rostral and caudal palatal shelves of hypomorph embryos at E12.5 and E14.5 by measuring the relative *CyclinD1* gene expression using qPCR. Student's *t*-test; error bars indicate±s.e.m. *P<0.05, **P<0.01, ***P<0.001. C, caudal palate; lnp, lateral nasal prominence; md, mandibular prominence; mx, maxillary prominence; np, nasal prominence; pits, nasal pits; R, rostral palate.

Split facial prominences and, thus, a widely opened face (i.e. midfacial cleft) were detected in *Cdk13^tm1d/tm1d^* embryos at E11.5, revealing also the development of telencephalon in the midline (Fig. 1A″). In *Cdk13^tm1a/tm1a^* embryos at the same embryonic stage, we detected widely set facial prominences (Fig. 1A′). Measurements of distances between individual facial prominences revealed a significantly greater ratio of nasal pits distances and head width in maxillary prominence and lateral nasal prominence levels in both mutant genotypes (Fig. 2A-C). Widely set facial prominences at earlier embryonic stages then progress at E12.5 to median cleft lip in *Cdk13^tm1a/tm1a^* embryos (Fig. 1B′,H′) but persisted in *Cdk13^tm1d/tm1d^* embryos, leading to the development of midfacial cleft (Fig. 1B″,H″). Delayed development of the palatal shelves (Fig. 1E′,K′,K″) with cleft nasal septum (Fig. 1K′,K″) was detected in both mutant genotypes. Later, at E13.5 and E14.5, *Cdk13^tm1a/tm1a^* embryos displayed thinner lips with increased distance between (Fig. 1F′,I′,G′J′) and delayed development of the palatal shelves (Fig. 1F′,L′,G′,M′), while *Cdk13^tm1d/tm1d^* embryos exhibited a severe midfacial cleft (Fig. 1C″,I″,D″,J″) and persisting nasal septum cleft (Fig. 1L″,M″). Less severe anomalies were detected in the caudal palate area, in which underdeveloped palatal shelves were the most prominent feature, especially in *Cdk13^tm1d/tm1d^* embryos (Fig. S1E-G). For *Cdk13^tm1a/tm1a^* embryos observed at the latest embryonic stage (i.e. E16.5), improperly fused lips in the midline as well as cleft palate persisted within the rostral and caudal areas, accompanied with necrotic tissue that was visible within all structures (Fig. S1B-B″,D,D′). This confirmed embryonic lethality after E15.5, which had also been noticed previously (Nováková et al., 2019).

Based on craniofacial morphometrics, heads and individual facial prominences are generally smaller in *Cdk13*-deficient embryos. Thus, we quantified gene expression of *CyclinD1*, a gene responsible for cell cycle progression from G1 to S phase and used it as a marker of proliferation to see if there is a change in proliferation rates. Compared with control embryos, *CyclinD1* expression was detected to be upregulated in almost all tissues isolated from facial prominences at E11.5 and E12.5 of *Cdk13*-deficient embryos (Fig. 2E). Although the palatal shelves in *Cdk13*-deficient animals were generally smaller and exhibited a different shape, there were no significant changes detected in *CyclinD1* expression in the palatal shelves of E12.5 *Cdk13^tm1a/tm1a^* embryos. A slight downregulation of *CyclinD1* was detected in both palatal regions isolated from E14.5 *Cdk13^tm1a/tm1a^* mice (Fig. 2E). Additional immunostaining for Ki-67 and TUNEL assays confirmed no significant changes in proliferation and apoptosis within the developing palatal shelves of *Cdk13^tm1a/tm1a^* embryos (Fig. S2A-C).

### Expression of *Cdk13* mRNA is dispersed through developing facial regions

**Fig. 3.**
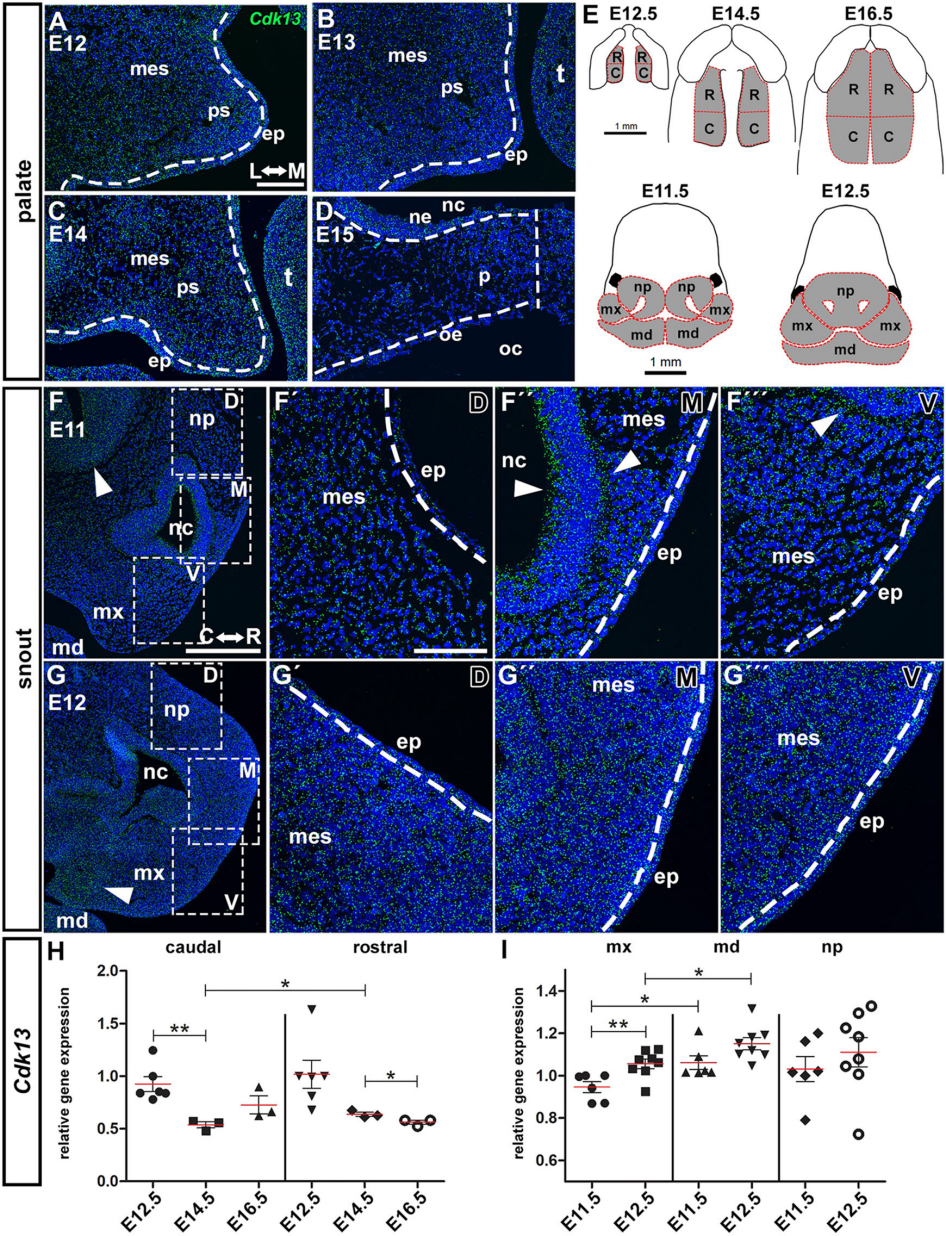
**Physiological gene expression of *Cdk13* within developing palatal tissues and snout detected by RNAScope and quantified by qPCR.** (A–D) *Cdk13* (green) gene expression (RNA levels) in the developing palatal shelves of WT mice between E12 and E14, and in the fused secondary palate on transversal sections at E15. White dashed lines separate mesenchyme from epithelium. Vertical white dashed line (D) highlights the secondary palate midline at E15. L↔M, lateral↔medial. (E) Schematics showing how individual tissues were dissected to quantify *Cdk13* gene expression. RNA expression was quantified in tissues dissected from rostral (R) and caudal (C) palatal shelves or palates at E12.5, E14.5 and E16.5 and in tissues dissected from mx, md and np at E11.5 and E12.5. (F,G′′) Lower power magnification of *Cdk13* gene expression in the developing snout of WT embryos at E11 and E12. Boxed areas in F and G are shown at higher magnification in panels F′-F′′′ and G′-G′′′, respectively, which show dorsal (D); middle (M) and ventral (V) areas as indicated in the top right of each image. Arrowheads indicate higher *Cdk13* signal intensity in the developing forebrain (F) and nasal epithelium (F″,F′″) at E11, and the developing maxillary prominence (G) at E12. Dashed lines separate mesenchyme and epithelium. Nuclei were counterstained with DAPI. C↔R, caudal↔rostral. (H) Quantification of *Cdk13* gene expression after using qPCR in palatal shelves at E12.5, E14.5 and E16.5. (I) Quantification of the *Cdk13* gene expression by qPCR in mx, md and np at E11.5 and E12.5. Unpaired two-tailed Student’s *t*-test; *P<0.05; **P<0.01; ***P<0.001. C, caudal; ep, epithelium; md, mandibular prominence; mes, mesenchyme; mx, maxillary prominence; nc, nasal cavity; ne, nasal epithelium; np, nasal prominence; oc, oral cavity; oe, oral epithelium; p, palate; ps, palatal shelf; R, rostral; t, tongue. Scale bars: 100 µm (A-D, F′-G‴), 200 µm (F′,G).

As we observed distinct changes in facial and palatal morphogenesis in *Cdk13*-deficient embryos, we asked if there is localized expression of *Cdk13* mRNA within certain areas during craniofacial development. We evaluated gene expression of *Cdk13* on frontal sections to reveal possible differences in its distribution along the labio-lingual axis of the palatal shelves. In embryos between E12 and E14, the *Cdk13* signal was spread evenly within the palatal mesenchyme and epithelium of the palatal shelves (Fig. 3A-C). Later, at E15, we detected increased expression of *Cdk13* in the palatal mesenchyme, close to the region of fusion in the craniofacial midline (Fig. 3D). In the developing snout, intensity of the *Cdk13* signal was similar in the surface epithelium and mesenchyme. A visibly stronger signal was detected in the forebrain (Fig. 3F) and nasal epithelium at E11 (Fig. 3F″,F″′), and in the maxillary mesenchyme at E12 (Fig. 3G). At later stages (E13, E14), *Cdk13* mRNA was evenly spread in the developing snout in both mesenchyme and epithelium (Fig. S3). Quantification of relative *Cdk13* expression by qPCR revealed decreasing levels in the palatal area throughout the development (Fig. 3H) and increasing levels in the maxillary, mandibular and nasal prominences between E11.5 and E12.5 (Fig. 3I).

To uncover possible differences of *Cdk13* distribution along the rostral-caudal axis of the developing palate, we analyzed its expression in the sagittal sections. During earlier developmental stages (E11-E14), *Cdk13* was expressed in both rostral and caudal areas of the developing palatal shelves, with higher *Cdk13* signal intensity detected in the rostral region compared to that in caudal areas (Figs S4A-D and S5). Changes emerged in older embryos (E15-E16), where we observed strong expression of *Cdk13* in the palatal epithelium and adjacent mesenchyme, especially in the developing palatal rugae, both in the rostral and caudal regions (Fig. S4E,F).

### CDK13 is located in cellular outgrowths as well as long neural processes

Next, we asked how CDK13 protein is distributed in cells *in vitro*. We analyzed its expression in cultured mouse embryonic fibroblasts (MEFs) and cells derived from DRGs (dorsal root ganglia) of adult mice as these cell types typically form long cytoplasmic processes.

**Fig. 4.**
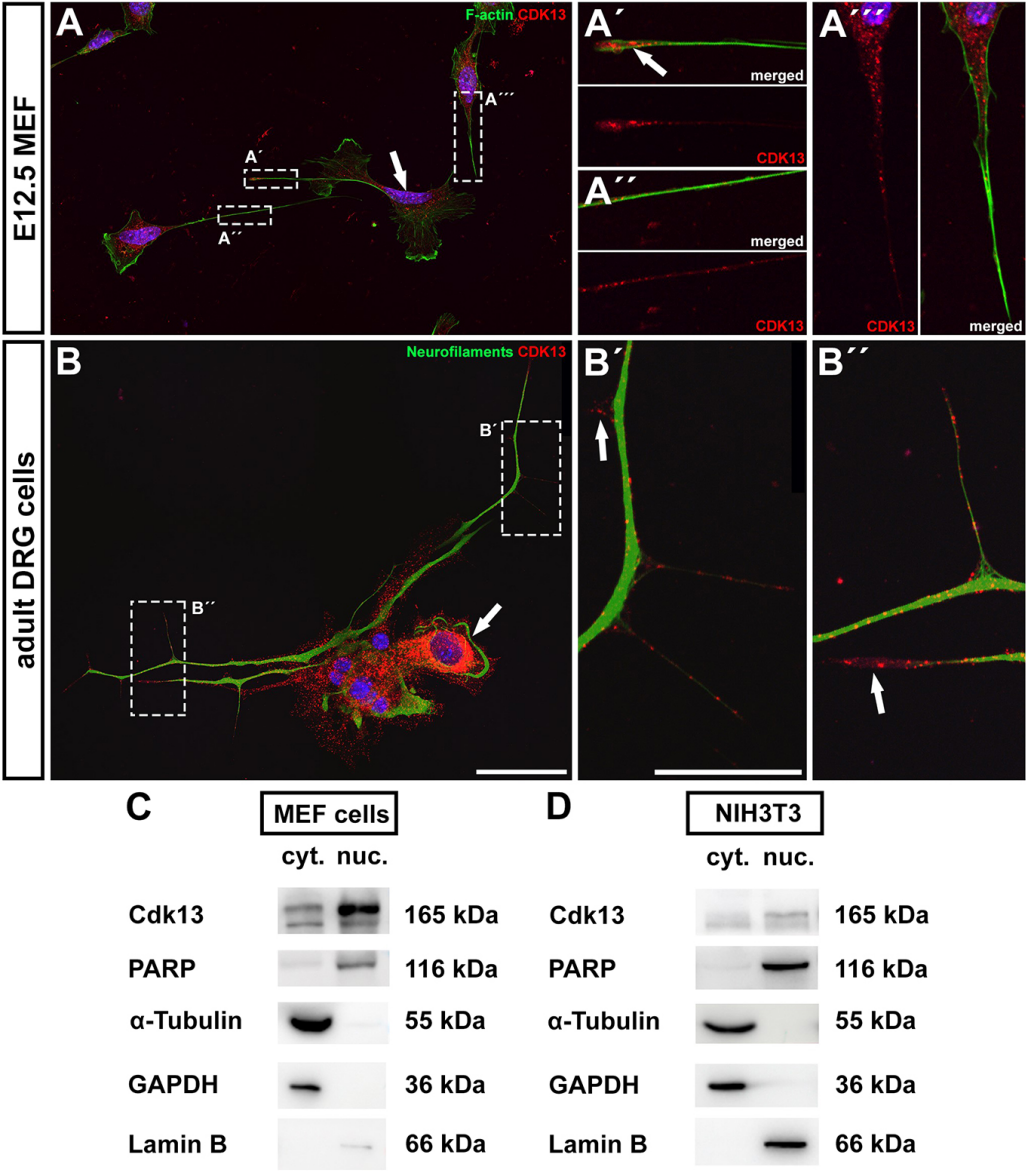
**CDK13 cellular localization using immunocytochemistry and western blotting.** (A-A‴) Immunofluorescence images of mouse embryonic fibroblasts (MEFs) isolated from embryonic bodies at E12.5, stained for CDK13 (red) and F-actin (green). Boxed areas indicate details of cellular outgrowths and are shown magnified in A′-A′′′ as indicated. Arrows point to the nuclear area (A) and cellular outgrowths (A′) with increased CDK13 expression. (B-B′′) Immunofluorescence images of primary cells isolated from dorsal root ganglia of adult mice stained for CDK13 (red) and neurofilaments (green; anti-neurofilament antibody 2H3). Boxed areas indicate neurite outgrowths and are shown magnified in B′-B′′ as indicated. Arrows point to the nuclear area (B) and neurite outgrowths (B′,B′′) with CDK13 expression. Nuclei were counterstained with DAPI. Scale bars: 50 µm (A,B), 20 µm (A′-B′′). (C,D) Western blotting for CDK13 in cytoplasmic (cyt.) and nuclear (nuc.) extracts from primary MEF cells (C) and NIH3T3 mouse fibroblasts (D), showing CDK13 levels in both nuclear and cytoplasmic fractions. Lamin B and PARP1 were used as positive loading controls for the nuclear fraction; α-tubulin and GAPDH were used as positive loading controls for the cytoplasmic fraction.

In MEF cells, CDK13 was localized to the nuclear area and enriched in the cytoplasm around the nucleus (Fig. 4A). However, CDK13 protein was also detected within cellular outgrowths (Fig. 4A′-A″′), including their most apical tips, where it was located together with actin filaments (Fig. 4A′). In cells isolated from DRGs, CDK13 was detected in the nuclear area, with highest levels detected in the cytoplasm adjacent to nuclei (Fig. 4B). As observed in cultured MEFs, we also detected CDK13 within long neural processes along the neurofilaments that were forming the outgrowths (Fig. 4B′,B″). CDK13 in cytoplasm as analyzed by immunofluorescence staining was further evaluated by cellular fractionation followed by western blotting for CDK13. As expected, CDK13 was detected in the nuclear fraction of primary MEF cells; however, significant levels of CDK13 were also detected in the cytoplasmic fraction (Fig. 4C). Moreover, the mouse fibroblast cell line NIH3T3 was used to assess the subcellular localization of CDK13, and CDK13 was found in both cytoplasmic and nuclear cellular fractions, with higher levels in the nuclear fraction (Fig. 4D).

Hypomorphic mutation causes production of a truncated form of CDK13, with preserved N-terminal domain (Nováková et al., 2019). This form of CDK13 aggregated in the cellular processes together with deposits of F-actin in E12.5 *Cdk13^tm1a/tm1a^* MEFs (Fig. S6A). Similarly, aggregates of truncated CDK13 were located in protrusions of cells isolated from E12.5 *Cdk13^tm1a/tm1a^* DRGs (Fig. S6B). Additionally, live cell imaging of MEF cells revealed different numbers of cellular protrusions per cell in *Cdk13^tm1d/tm1d^* cells (Fig. S6C, top) compared to control cells. This was associated with the more-stretched morphology of cultured *Cdk13^tm1d/tm1d^* cells (Fig. S6D), their weaker adherence to culture plates and easier release from plates during trypsin digestion (data not shown). However, cellular velocity (Fig. S6C, bottom) and the percentage of the cellular surface being protruded (Fig. S6C, middle) showed no statistically significant difference between either genotype in MEF cultures.

This specific cellular localization of the CDK13 to cellular protrusions indicates a potential cytoplasmic role of the CDK13 protein. However, when we performed detection of ICC signal by polyclonal anti-CDK13 antibodies, we revealed a reduction of the CDK13-specific signal in nuclei (see arrowheads in Fig. S6E) but not in the cytoplasm surrounding the nuclei or the cellular protrusions of *Cdk13^tm1d/tm1d^* cells (Fig. S6E), indicating primary effect of *Cdk13* deficiency in nuclei.

### *Cdk13* deficiency results in development of hypoplastic cranial nerves

Development of the craniofacial region is closely associated with the development of cranial nerves. A large area is innervated by the trigeminal nerve comprising three main branches, i.e. the maxillary, mandibular and ophthalmic branch (Higashiyama and Kuratani, 2014). We detected complex spatial *Cdk13* gene expression in the developing maxillary nerve in cells that ensheath bundles of nerve fibers (Fig. S7A-E′). As the CDK13 has been demonstrated to regulate neurite outgrowth (Chen et al., 2014), we hypothesized that alteration of cranial nerve growth occurs during early craniofacial development in *Cdk13*-deficient animals.

**Fig. 5.**
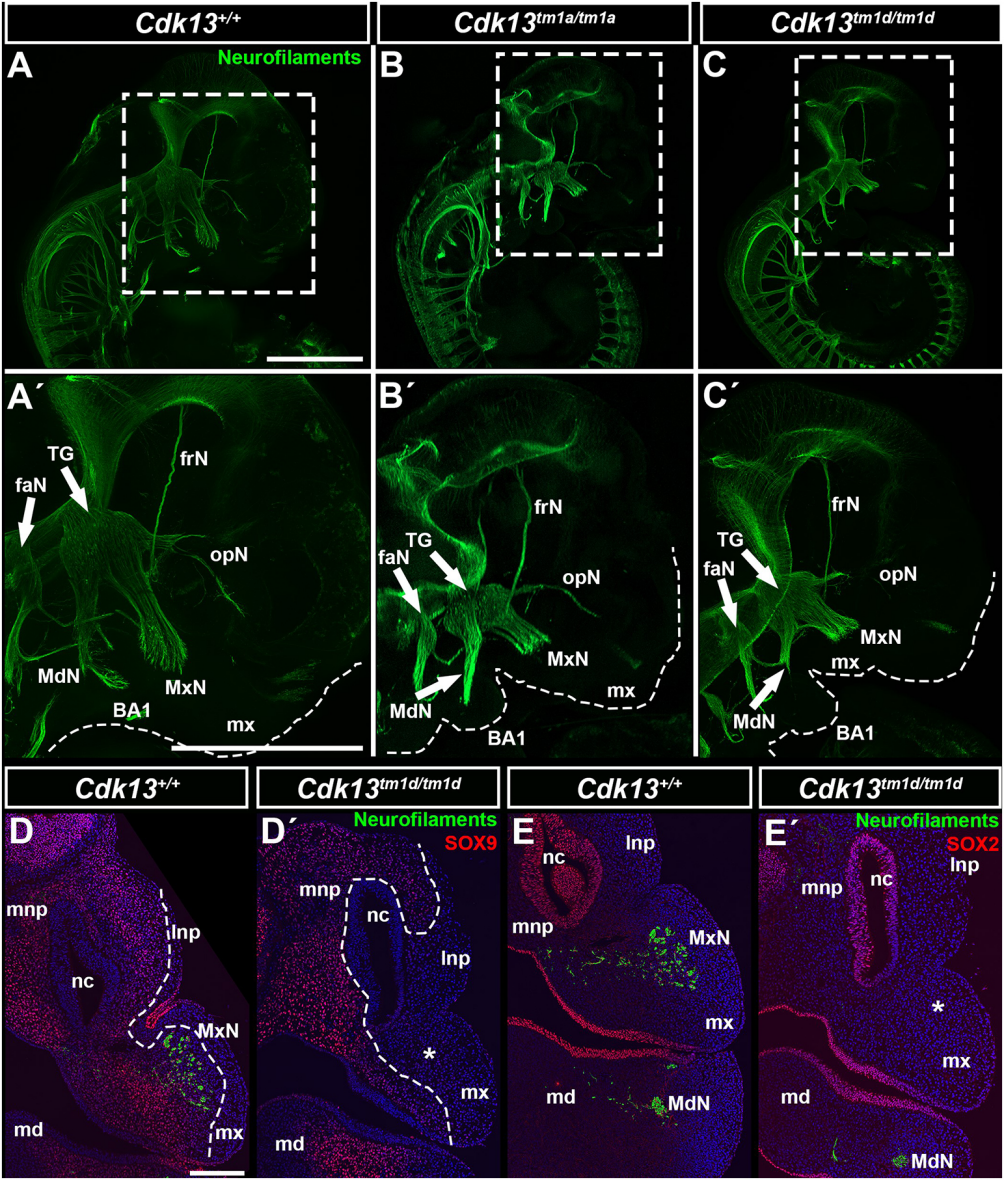
**Immunohistochemical detection of cranial nerves on whole-mount embryos and histological sections.** (A-C′) Whole-mount images stained to detect developing neurons (green) staining anti neurofilament antibody (anti-2H3) in WT, *Cdk13^tm1a/tm1a^* and *Cdk13^tm1d/tm1d^* embryos at E11.5. Dashed line rectangles highlight detail of the craniofacial region. Boxed areas are shown magnified in A′-C′. Detail displays the basis of the facial nerve, and divisions of the trigeminal nerve – mandibular, maxillary and ophthalmic nerves, including the frontal branch nerve of the ophthalmic nerve. Dashed lines indicate the edge of the maxillary prominences and the first branchial arch (BA1). Note underdeveloped and hypoplastic all three divisions of the trigeminal nerve in *Cdk13^tm1a/tm1a^* (B′) and especially in *Cdk13^tm1d/tm1d^* (C′) embryos. (D-E′) Immunohistochemistry images stained to detect neurofilaments (green), as well as SOX9 and SOX2 (red) within frontal sections of E11.5 embryos. Missing maxillary nerves in *Cdk13*-deficient embryos are marked by asterisks in D′ and E′. Dashed lines in indicate areas of SOX9 expression in D and D′. Scale bars: 1 mm (A-C′); 20 µm (D-E′). BA1, branchial arch 1; faN, frontal and acoustic nerve; frN, frontal branch of the ophthalmic nerve; lnp, lateral nasal prominence; md, mandibular prominence; MdN, mandibular branch of the trigeminal nerve; mx, maxillary prominence; MxN, maxillary branch of the trigeminal nerve; nc, nasal cavity; opN, ophthalmic branch of the trigeminal nerve; TG, trigeminal ganglion.

First, we visualized outgrowth of cranial nerves by whole-mount immunohistochemistry analysis of neurofilaments to uncover possible alterations of general morphology of trigeminal nerve and associated nerves. Alterations regarding the outgrowth of several cranial nerves were detected in *Cdk13^tm1a/tm1a^* and *Cdk13^tm1d/tm1d^* embryos (Fig. 5B,C′), with obvious hypoplasia of maxillary, mandibular and ophthalmic nerves, which were reduced in length. These nerves originate from the trigeminal ganglion (TG), which we also found to be abridged in the mutant embryos (Fig. 5B′,C′). Only the frontal nerve (frN) was not morphologically altered (Fig. 5B′,C′). This confirms that *Cdk13* deficiency negatively affects neurogenesis during embryonic development.

As observed by using whole-mount immunohistochemistry, changes of nerve protrusions were even more obvious on transversal sections of E11.5 *Cdk13^tm1d/tm1d^* embryos. In the rostral region, we observed missing maxillary nerves in the developing maxillary prominence (Fig. 5D′,E′; Fig. S8A′,B′), hypoplastic MnNs in the mandibular prominence (Fig. 5E′) and reduced size of the TG (Fig. S7F,G) confirming anomalies regarding nerve growth in *Cdk13*-deficient animals.

### Changes in the expression of key developmental genes and proteins during early craniofacial development of *Cdk13*-deficient embryos

**Fig. 6.**
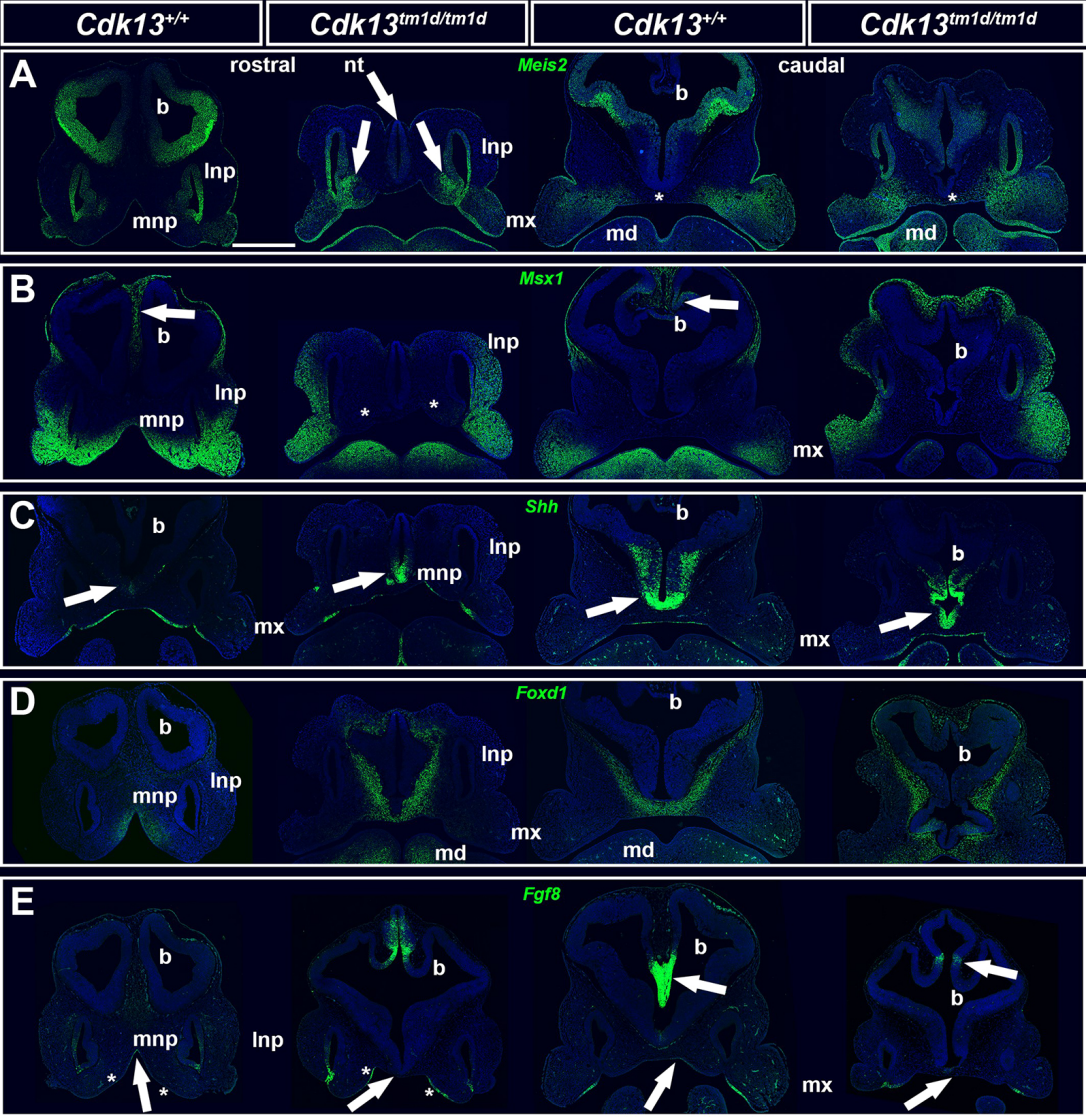
**RNAScope detection of *Meis2*, *Msx1*, *Shh*, *Foxd1* and *Fgf8* gene expression within frontal sections of WT and mutant mouse embryos at E11.5.** RNAScope fluorescence images showing changes of gene expression patterns in the maxillary prominence (mx), mandibular prominence (md), lateral nasal prominence (lnp) and medial nasal prominences (mnp) and the developing brain from WT (*Cdk13*^+/+^) and *Cdk13*-knockout (*Cdk13t^m1d/tm1d^*) embryos. (A) *Meis2* gene expression. Arrows point to *Meis2* expression in the mesenchyme of the np and to lack of rostral expression in the nt of mutant. Asterisks indicate caudal differences in *Meis2* expression within the median mesenchyme. (B) *Msx1* gene expression. Arrows point to *Msx1* expression within WT brain. Asterisks indicate lack of *Msx1* expression in the mnp of mutant. (C) *Shh* gene expression. Arrows point to changes in the pattern of expression domains within the developing brain in WT and mutant. (D) *Foxd1* gene expression. Note the enhanced expression of *Foxd1* in mesenchyme around the nt in the rostral area of the *Cdk13* knockout compared to only trace amount around the brain in the WT embryo. (E) *Fgf8* gene expression. Arrows point to changes in *Fgf8* expression pattern in the epithelium, covering the middle area of the presumptive oral cavity, and the brain. Asterisks indicate changes in *Fgf8* expression pattern in the epithelium, covering the lateral area of the presumptive oral cavity within the rostral region. Individual gene expression patterns are shown in green. For each set of images, rostral sections are shown in the first two panels (from left) and caudal sections are shown in the last two panels (from left). Scale bar: 500 µm. b, brain

We also detected alterations in the expression patterns of genes and proteins strongly associated with craniofacial development, and specifically enriched in mesenchymal (SOX9, *Msx1*, *Foxd1*) and epithelial structures (SOX2, *Meis2*, *Shh*) in this region. In E11.5 *Cdk13^tm1d/tm1d^* embryos, we observed very low levels of the neural crest marker SOX9 in the mesenchyme of lateral nasal prominences and maxillary prominences, including an area with undeveloped maxillary nerves (Fig. 5D′; Fig. S8A′). However, the pattern of SOX2, one of the regulators of neural cells differentiation and peripheral nervous system development (Adameyko et al., 2012), was similar to that in wild-type (WT) embryos, only missing in the area of the prospective maxillary nerve (Fig. 5E′; Fig. S8B′). Expression of *Meis2*, a gene involved in cranial neural crest development (Machon et al., 2015), was absent from the neural tube in the rostral area (Fig. 6A, see arrow at the top, indicating nt) and less expressed in the same tissue more caudally. Moreover, *Meis2* expression was detected caudally, close to the facial midline in knockout (Fig. 6A, asterisks) but not in WT mice. Conversely, *Meis2* was enriched in the mesenchyme of the rostral medial nasal prominences (Fig. 6A). *Msx1*, a key player in craniofacial development (Levi et al., 2006), was absent from the mesenchyme of the medial nasal prominences (asterisks) and also from the brain close to its midline (Fig. 6B). However, analysis of genes necessary for determination and development of facial primordia (Jeong et al., 2004), revealed that expression of *Shh* was enhanced in the ventral neural tube within the rostral region but also, to a similar extend, in the developing brain more caudally (Fig. 6C, arrows). Its altered expression pattern in the epithelium covering the forming stomodeal cavity was detected rostrally (Fig. 6C). Moreover, enhanced expression of *Foxd1*, which is expressed downstream of *Shh*, in the developing mandibular prominences points to altered development of the lower jaw (Fig. 6D). Finally, expression of *Fgf8* was absent in the epithelium of the developing primitive oral cavity close to the head midline (Fig. 6E, arrows and asterisks) and its expression was reduced in the developing brain (Fig. 6E, asterisks). This confirmed that *Cdk13* deficiency leads to deregulation of these key developmental genes already in early craniofacial development, subsequently resulting in severe facial clefting.

### *Cdk13* deficiency alters expression of neurogenesis-specific genes and key morphogenic proteins in the developing secondary palate

**Fig. 7.**
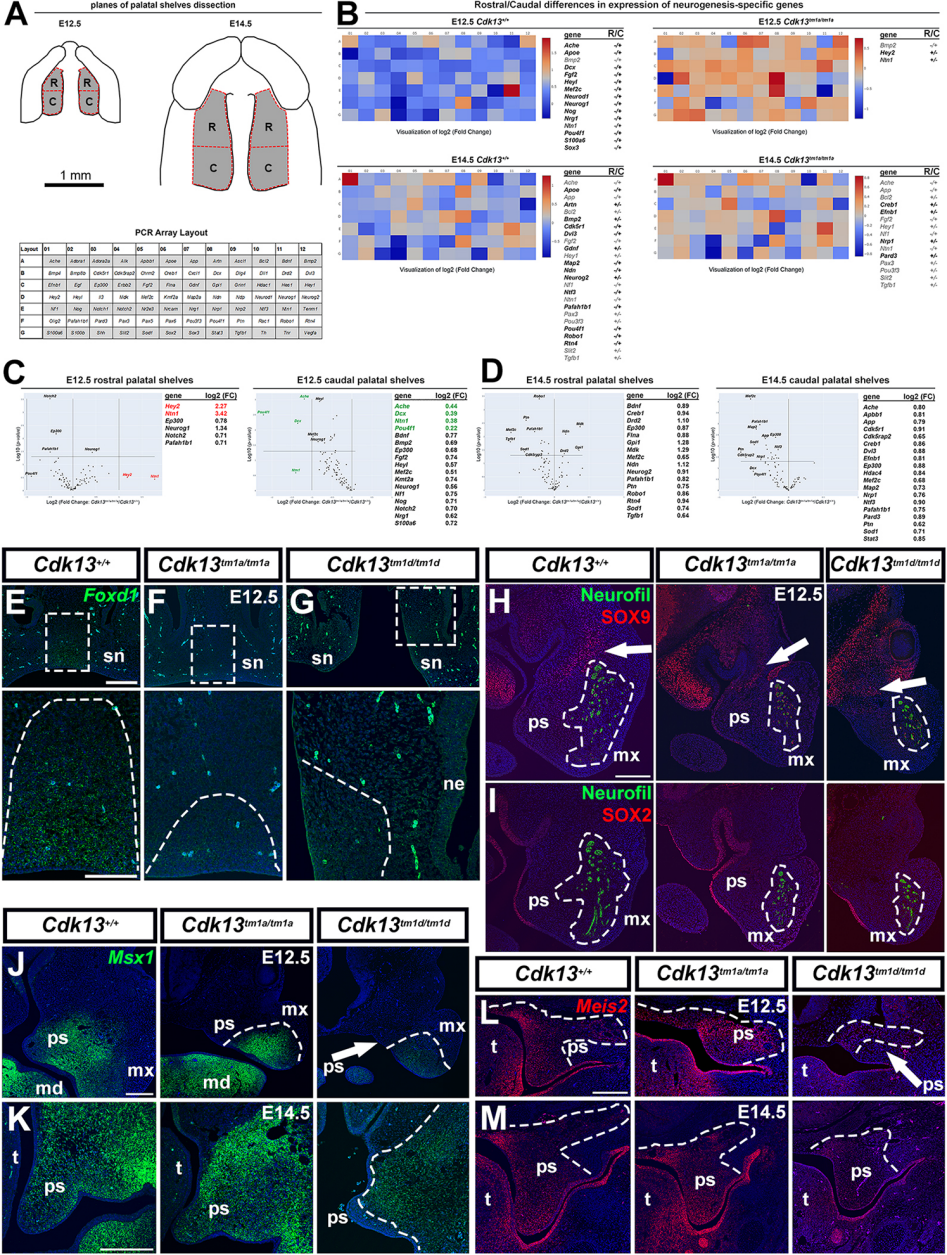
**Quantification of neurogenesis-associated gene expression in the palatal shelves of WT and *Cdk13^tm1a/tm1a^* mice, and *in situ* gene and protein expression within sections obtained from WT and mutant mice.** (A-D) PCR Array results for mouse neurogenesis-specific genes performed separately on tissues dissected from rostral and caudal palatal shelves of WT and *Cdk13^tm1a/tm1a^* embryos at E12.5 and E14.5. (A) Schematics showing dissected rostral (R) and caudal (C) regions of the palatal shelves at E12.5 and E14.5, and the layout of the PCR array analysis of individual detected genes. (B) Heat maps and lists of genes presenting rostral/caudal differences in the expression of neurogenesis-specific genes in control (WT) embryos (left panels, top and bottom) and *Cdk13^tm1a/tm1a^* embryos (right panels, top and bottom). Genes listed in black are enriched either in the rostral or caudal palatal shelves. Genes listed in grey are expressed in the palatal regions in similar extent. Notice the changed ratio of the gene expression polarity when comparing palatal shelves of WT and *Cdk13*-deficient mice, i.e. differences between genes expressed either in the rostral or caudal palatal shelves are much less in *Cdk13*-deficient tissues. (C,D) Volcano plots and log2-fold changes individually displaying significantly deregulated expression of neurogenesis-specific genes in *Cdk13*-deficient palatal shelves at E12.5 (C) and E14.5 (D). Genes listed in red are strongly upregulated and genes listed in green text are strongly downregulated in *Cdk13*-deficient embryos compared to control embryos. *P* values were calculated based on Student's *t*-test of replicate 2^(- Delta C_T_) values for each gene in control and test groups. *P*-value calculation used was based on parametric, unpaired two-sample equal variance, two-tailed distribution. (E-G) RNAScope detection of *Foxd1* (green) *in situ* gene expression in sections of the rostral snout region of WT and mutant embryos as indicated, at E12.5. Boxed areas are shown magnified below the respective panel. Dashed lines in magnified images outline areas of *Foxd1* expression (H-I) Fluorescence images of rostral snout sections as described for panels E-G, stained for SOX9 (red) or SOX2 (red) and neurofilaments (green; anti-neurofilament antibody 2H3). Arrows point to regions enriched with SOX9-positive cells. Dashed lines surround neurofilaments, indicating developing maxillary nerves. (J-K) RNAScope detection of *Msx1* (green) *in situ* gene expression in sections of the rostral palate region of WT and mutant embryos as indicated, at embryos at E12.5 and E14.5. Dashed lines outline *Msx1-*positive regions. (L-M) RNAScope detection of *Meis2* (red) *in situ* gene expression in sections of the caudal palate region of WT and mutant embryos at E12.5 and E14.5. Dashed lines outline *Meis2-*positive regions. Scale bars: 200 µm (E-G top row; H-M), 100 µm (E-G magnified images, bottom row). md, mandibular prominence; mx, maxillary prominence; ne, nasal epithelium; ps, palatal shelves; sn, snout; t, tongue.

To further evaluate alterations in the expression of genes relevant to neurogenesis during palatogenesis in *Cdk13^tm1a/tm1a^* embryos, we used PCR array analysis to simultaneously investigate the expression profile of 84 neurogenesis-specific genes. In separately dissected tissues from rostral and caudal palatal shelves (Fig. 7A), we first compared expression of neurogenesis-specific genes in rostral and caudal palatal regions (Table S1-S4).

Of all genes analyzed this way (Fig. 7A), 15 genes (*Ache*, *Apoe*, *Bmp2*, *Dcx*, *Fgf2*, *Heyl*, *Mef2c*, *Neurod1*, *Neurog1*, *Nog*, *Nrg1*, *Ntn1*, *Pou4f1*, *S100a6* and *Sox3*) were found to be significantly enriched in caudal compared to rostral palatal shelves in control (WT) embryos at E12.5 (Fig. 7B, top left panel). The same comparison between rostral and caudal palatal shelves in *Cdk13^tm1a/tm1a^* embryos at E12.5 determined a preserved gene expression ratio only for *Bmp2* – probably caused by the downregulation of expression of caudal palatal shelve-dominant genes (*Ache*, *Dcx*, *Ntn1*, *Pou4f1*, *Bdnf*, *Bmp2*, *Ep300*, *Fgf2*, *Heyl*, *Mef2c*, *Kmt2a*, *Neurog1*, *Nf1*, *Nog*, *Notch2*, *Nrg1* and *S100a6*) in the caudal palatal shelves (Fig. 7C, right panel). By contrast, a change in ratio was detected for *Hey2* and *Ntn1*, whose expression was – conversely – enriched in the rostral palatal shelves (Fig. 7B, top right panel), caused by extreme upregulation of both genes in mutant embryos (Fig. 7C, left panel).

In control embryos at a later stage, i.e. at E14.5, ten genes were enriched in rostral palatal shelves (*Artn, Bcl2*, *Bmp2*, *Gdnf*, *Hey1*, *Neurog2*, *Pax3*, *Pou3f3*, *Slit2* and *Tgfb1*) and 15 genes in caudal palatal shelves (*Ache*, *Apoe, App, Cdk5r1, Dvl3, Fgf2, Map2, Ndn, Nf1, Ntf3, Ntn1, Pafah1b1, Pou4f1, Robo1* and *Rtn4*), (Fig. 7B, bottom left panel). Again, the same comparison between rostral and caudal palatal shelves, this time in E14.5 *Cdk13^tm1a/tm1a^* embryos, revealed a preserved gene expression ratio for 11 genes (*Ache*, *App*, *Bcl2*, *Fgf2*, *Hey1*, *Nf1*, *Ntn1*, *Pax3*, *Pou3f3*, *Slit2* and *Tgfb1*) in E14.5 *Cdk13^tm1a/tm1a^* embryos. Generally, deregulation of the neurogenesis-specific genes in the E14.5 palatal shelves was not as substantial (Fig. 7D) as was in case for E12.5 (Fig. 7C). But, in both observed embryonic stages, gene expression was generally downregulated in the caudal compared to the rostral region, whereas in the rostral region both up- and downregulation of gene expression was detected (Fig. 7C,D).

Moreover, altered expression patterns of palatal genes and proteins were detected later at E12.5 and E14.5. *Cdk13*-deficiency leads to noticeable reduction of *Foxd1* expression in the mesenchyme of snout midline (Fig. 7F,G), *Msx1* reduction in the rostral palatal shelves (Fig. 7J,K) and also reduction of *Meis2* in the caudal palatal shelves (Fig. 7L,M), especially at E12.5. Levels and distribution of both SOX9 and SOX2 were similar to those observed for WT animals (Fig. 7H,I).

### *Cdk13* deficiency results in changes of RNAPII occupancy within promoters of *Pou4f1* and *Ntn1*

To address the effect of CDK13 depletion on transcription, we performed chromatin immunoprecipitation (ChIP) with an antibody specifically recognizing RNA polymerase II (RNAPII) and IgG as a negative control (Fig. S9). For two genes selected from the PCR array analysis (Fig. 7), i.e. *Pou4f1* and *Ntn1*, we decided to evaluate the presence of RNAPII within their promoter regions in MEF cells obtained from *Cdk13^+/+^* and *Cdk13^tm1d/tm1d^* mice. Increased levels of RNAPII within either promoter of *Pou4f1* and *Ntn1* were observed in comparison to IgG control (Fig. S9A,B). In the case of the *Pou4f1* promoter, we detected less RNAPII in MEF cells obtained from *Cdk13^tm1d/tm1d^* compared to those obtained from *Cdk13^+/+^* mice (Fig. S9A). In contrast, RNAPII was associated more with the *Ntn1* promoter in *Cdk13^tm1d/tm1d^* MEFs than in *Cdk13^+/+^* MEF cells (Fig. S9B). We then decided to compare the presence of RNAPII on these promoters as a percentage of RNAPII compared to IgG control. A slight decrease of RNAPII was observed for the *Pou4f1* promoter in *Cdk13^tm1d/tm1d^* MEF cells compared with that in *Cdk13^+/+^* MEFs (Fig. S9C). In contrast, higher occupancy of RNAPII was demonstrated for the *Ntn1* promoter in *Cdk13^tm1d/tm1d^* MEFs when compared with *Cdk13^+/+^* MEFs (Fig. S9D). Although the statistical difference for RNAPII occupancy at these promoters was not significant, the obtained observation correlates with decreased and increased mRNA levels of *Pou4f1* and *Ntn1* genes as detected by using PCR expression screen (Fig. 7). However, future research regarding these findings will be necessary.

### Chemical inhibition of the CDK13/CDK12 leads to embryonic facial malformations and altered neurite outgrowth

To confirm the effect of CDK13 deactivation on craniofacial structure formation, we used the synthetic CDK13 inhibitor THZ531 to treat early maxillary prominences of *Gallus gallus* embryos. It is necessary to note that THZ531 is also targeting CDK12, which shares several functions with CDK13 and, in developmental processes and under certain conditions, they can replace each other. Therefore, the usage of this inhibitor should prevent also compensatory mechanisms induced by CDK13 deficiency.

**Fig. 8.**
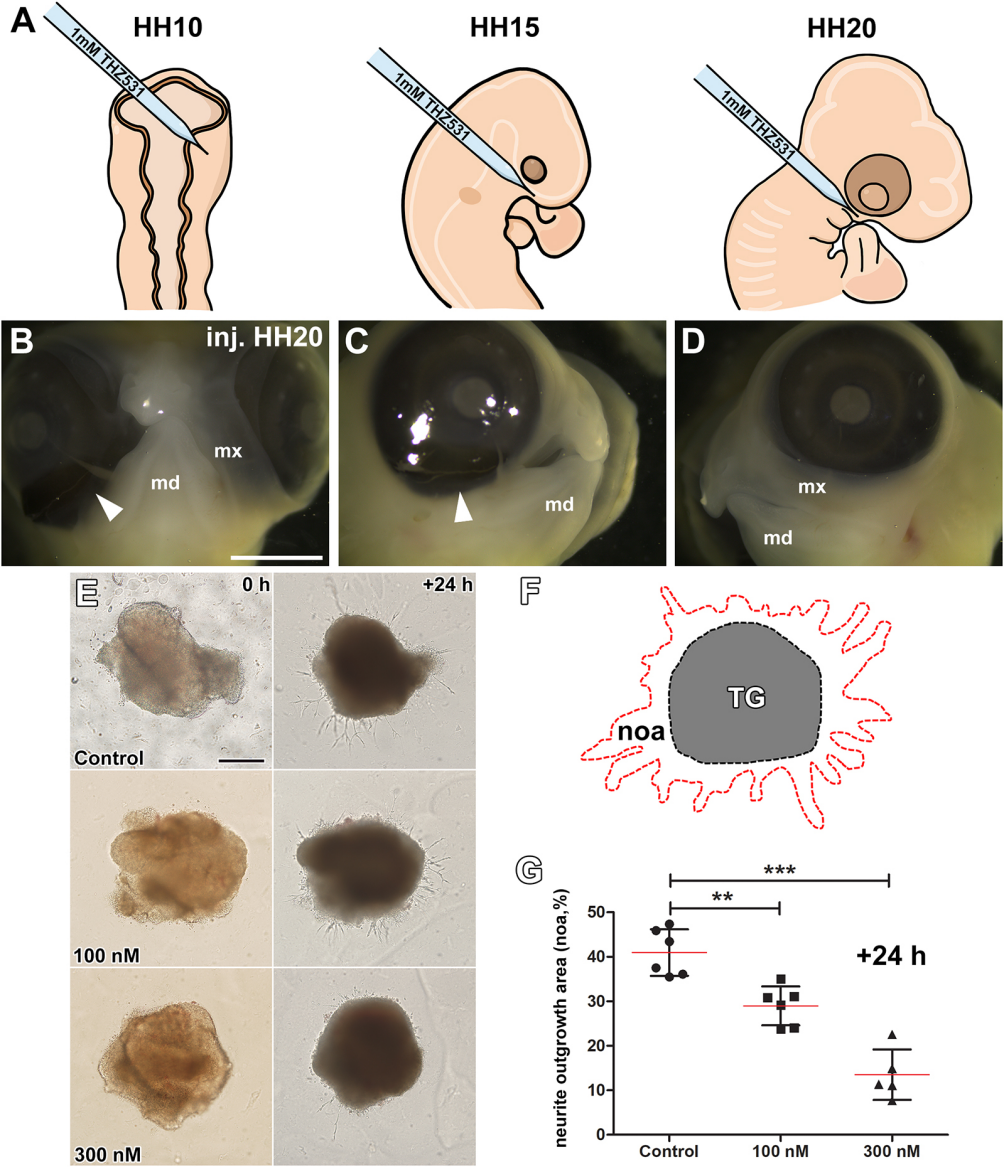
**Chemical inhibition of CDK12/CDK13 leads to embryonic facial malformations and altered neurite outgrowth in chicken and mouse.** (A) Schematics showing the injection side of the CDK12/CDK13 inhibitor THZ531 to the maxillary prominences in chicken at HH10, HH15 or HH20 followed by 4-6 days of incubation. (B-D) Macroscopic images showing the ventral and lateral regions of embryonic chicken heads that had either been injected (on the right side of the head) with THZ531 at HH20 (B,C) or not (D). White arrowheads indicate missing maxillary prominence in the ventral (B) and lateral (C) regions after inhibition of CDK12/CDK13. This anomaly is only present in heads that had been injected with THZ531 (D). Scale bar: 3 mm. md, mandibular prominence; mx, maxillary prominence. (E) General morphology of *ex vivo* trigeminal ganglia explants from E12 WT mouse embryos that had either been injected with THZ531 (100 nM or 300 nM) or not (Control). Images were taken at the beginning (0 h) and the end (+24 h) of cultivation. Notice the different length and number of neurite outgrowths under different conditions. Scale bar: 200 µm. (F) Schematic showing the areas used to measure the differences in neurite outgrowth area (noa; surrounded by red dashed line) from the trigeminal ganglion (TG; grey area surrounded by black dashed line). (G) Bar graph showing the neurite outgrowth area (in %) in WT trigeminal mouse ganglia cultivated for 24 h without (control), or with 100 nM or 300 nM of the CDK12/CDK13 inhibitor THZ531. Unpaired two-tailed Student’s *t*-test; error bars indicate ±s.e.m.; **P<0.01,***P<0.001. Replicates: Control (*n*=6), 100 nM (*n*=6), 300 nM (*n*=5).

We injected 1 mM THZ531 into the craniofacial mesenchyme at Hamburger–Hamilton (HH) developmental stage 10 (HH10), the postoptic region at HH15 and into maxillary prominences at HH20. Then, chicken embryos were allowed to develop for 4-6 days (Fig. 8A). This led to the absence or deficiency of maxillary prominences, resulting in the unilateral cleft lip (Fig. 8B,C). Injection of THZ531 at earlier stages resulted in a stronger phenotype with high deficiency in craniofacial tissues (Fig. S10).

Furthermore, the effect of *Cdk13* deficiency on cranial nerve development as shown earlier (Fig. 5) was additionally tested by chemical inhibition of CDK13 (together with CDK12) in functional *ex vivo* cultivation experiment using TG explants (Fig. 8). TGs were dissected from E12 WT mouse embryos and cultured, while culture medium was supplemented with THZ531 (100 nM or 300 nM). A significant reduction in the formation of neurite outgrowths was observed in TGs when cultured with different concentrations of the CDK13 inhibitor THZ531 compared to control group (Fig. 8E-G), revealing the importance of both CDK13 and CDK12 in development of cranial nerves.

## DISCUSSION

### CDK13 controls craniofacial morphogenesis by regulating the expression of genes that are part of signalling pathways important during development

Here, we determined a key contribution of CDK13 to craniofacial morphogenesis and neurogenesis, where the phenotype of *Cdk13-*deficient animals included a smaller head and disturbed facial morphology, including facial clefts*.* In our mutants, we found altered expression patterns of *Msx1*, *Meis2*, *Shh*, *Foxd1* and *Fgf8* in craniofacial structures. Downregulation or upregulation of these genes or their downstream targets causes similar phenotypic craniofacial phenotypes, including facial clefts or reduced growth of facial nerves (Levi et al., 2006; Machon et al., 2015; Jeong et al., 2004). Pronounced similarities in the morphology of craniofacial structures with *Cdk13*-knockout embryos has previously been observed in mice embryos with reduced functional expression levels of *Fgf8.* These animals also exhibited abnormal midline separation with nasal prominences set widely apart (Griffin et al., 2013). Such morphological changes indicated insufficiency in the development of neural crest cells (NCCs), from which all these affected craniofacial structures originate – which we confirmed by identifying reduced protein expression of the NCC marker SOX9 in facial prominences of *Cdk13*-deficient animals. Moreover, we detected significant downregulation of *Mef2c* (in all the palatal shelve regions, except rostral E12.5 palatal shelves). Its conditional downregulation, specifically in NCCs, has previously been found to result in craniofacial anomalies and neonatal lethality (Verzi et al., 2007).

### Anomalies in craniofacial morphogenesis and neurogenesis as a result of disrupted CDK-associated signaling

Similar to patients with mutations in *CDK13* or genes encoding other associated proteins, such as cyclin M, cyclin K, CDK10 (Colas, 2020) and CDK5RAP2 (Yigit et al., 2015), *Cdk13*-deficient mice produce neurogenic anomalies. These patients exhibit not just craniofacial anomalies but also affected neural tissues. Mutation in *cyclin M* results in optic nerve hypoplasia, mutation in *cyclin K* led to developmental delay and intellectual disabilities, and mutations in *CDK10* cause intellectual disabilities connected with language and learning disorders (Colas, 2020). Patients with loss-of-function mutation of *CDK5* display craniofacial anomalies other than facial clefts (short forehead, full cheeks, micrognathia), and also suffer of lissencephaly, cerebellar hypoplasia, microcephaly, intellectual disabilities, speech delay or autistic features (Colas, 2020), similar to patients with *CDK13* mutations (Hamilton and Suri, 2019).

CDK5 has a specific role in neural tissues physiology and regulates several neural processes, such as axonal transport, migration and synaptic vesicle endocytosis, and is also important for regulation of axon and neurite outgrowth (Shah and Rossie, 2018). Its knockout leads to perinatal lethality in mice, connected with deficient neuronal migration and impaired axonal transport of neurofilaments (Ohshima et al., 1996). Importantly, *in vitro* experiments using cortical neurons has demonstrated the role of CDK13 and also CDK12 in the regulation of neurite outgrowth through a common signaling pathway that involves modulation of *Cdk5* at RNA level (Chen et al., 2014). In our *Cdk13*-deficient models, we uncovered significant downregulation of genes encoding CDK5 regulatory subunits, such as of *Cdk5rap2* and *Cdk5r1*, in the caudal palatal shelves at E14.5, confirming the CDK13-CDK5 functional association observed in *in vitro* experiments (Chen et al., 2014). Moreover, *CDK5RAP2* mutations in human patients cause Seckel syndrome, manifested by microcephaly and cognitive problems (Yigit et al., 2015), and *CDK5R1* was proposed to be a candidate gene in patients affected by NF1 microdeletion syndrome, which mutations lead to non-syndromic intellectual disability and undefined facial deformities (Venturin et al., 2006).

In this study, we found the CDK12/CDK13 inhibitor THZ531 to inhibit neurite outgrowths from the TG (Fig. 8E,G). Even though one could argue that the observed effect might be a result of CDK12 and CDK13 together, it is broadly accepted, that inhibition of CDK12 primarily targets DNA-damage response (DDR) pathways and pre-replication complex assembly (Pilarova et al., 2020). The process of neurite outgrowth takes place in nondividing differentiated cells of the TG; therefore, it is unlikely that altered outgrowth is the result of CDK12 inhibition due to blocking of the cell cycle in S-phase. Also, there is no significant impact of CDK12 inhibition of the DDR pathway since cells with lesser outgrowth of neurites do not exhibit any signs of DDR stress. Thus, the documented reduction in neurite outgrowth is likely a result of CDK13 inhibition through a different mechanism or, alternatively, a combinatory effect of CDK12 and CDK13 inhibition through regulation of CDK5 function in neural cytoskeleton organization; however, this needs further experimental evidence.

### Effect of CDK13 on neurogenesis-specific gene expression and cranial nerve development

CDK13 is ubiquitously expressed in the murine snout, developing palatal shelves and cranial nerves and its mutation leads to prominent facial clefts. Craniofacial structures, including the palate, develop thanks to large contribution of NCCs. Growing peripheral nerves and glial cell types originate mostly from migrating NCCs and Schwann cell precursors (SCPs) (Furlan and Adameyko, 2018) but there are few exceptions, among them sensory neurons, which innervate maxillary region including the palate. These neurons originate in mesencephalic trigeminal nucleus in the CNS, and their development is regulated by FGF8 and Pou4f1 (Hunter et al., 2001), in agreement to that study, expression of both genes was detected altered in *Cdk13*-deficient embryos by us.

Along with these findings, we detected deregulated expression of genes that are typically associated with the TG in the developing palatal shelves. Expression of *Nrg1* (Meyer et al., 1997), a gene encoding an EGF receptor ligand, which in turn induces axon growth (Onesto et al., 2021) was downregulated in the palatal shelves at E12.5. Interestingly, *Nrg1* deficiency in mice also leads to maxillary dysmorphology (Waddington et al., 2017), an alteration from which palatal shelves are developing. Another gene usually detected in the TG is the neural transcription factor *Neurog1* that previously has also been associated with the palatal shelves (Visel et al., 2007). Its downregulation in TGs was observed as a result of *Pou4f1* downregulation (Lanier et al., 2009). Similarly, we found *Pou4f1* and *Neurog1* deregulated in palatal shelves of mice at E12.5. However, deregulation of *Neurog1* expression corresponded with expression changes of *Ntn1,* a gene encoding a secreted factor that is responsible for the guiding of developing peripheral motor axons (Serafini et al., 1996), and the proposed risk factor for development of the non-syndromic cleft lip and cleft palate (Leslie et al., 2017). Expression of both genes was highly upregulated in rostral palatal shelves and strongly downregulated in caudal palatal shelves, which indicates a tendency to rescue nerve growth to rostral regions of the developing face. Fewer nerves and their reduced development in the facial region of *Cdk13*-deficient embryos is further accompanied with significantly downregulated expression of the *Ache* gene in the caudal palatal shelves at E12.5. *Ache* encodes an acetylcholinesterase necessary for the degradation of acetylcholine, leading to termination of signal transduction at neuromuscular junctions, which is also important for neurite outgrowth and its elongation (Olivera et al., 2003).

In *Cdk13*-deficient animals, we also determined changes in the expression of genes involved in neuronal migration, possibly leading to alteration of nerve growth. One of such genes, whose expression was detected to be downregulated in all analyzed palatal shelve regions, is *Pafah1b1*. This gene encodes a non-catalytic subunit of an enzyme responsible for activation of GTPases and actin polymerization at the leading edge of locomoting neurons making it important for neural migration (Kholmanskikh et al., 2003). Its mutation in humans frequently results in lissencephaly and Miller–Dieker syndrome (Liu et al., 2021), a disease with very phenotypic manifestations similar to those of *Cdk13* deficiency, including growth delay and cerebral, cardiovascular, facial and limb anomalies (Yingling et al., 2003).

### Diverse levels of CDK13 action during embryonic development

CDK13 was originally discovered as a nuclear factor participating in the regulation of transcription. We were able to detect CDK13 in the nucleus by immunofluorescence and cellular fractionation; however, we also determined significant levels of CDK13 in the cytoplasm. This might seem a rather surprising observation but presence of CDK13 in the cytoplasm of various cancer cells has been already documented by another group (Wu et al., 2023). Importantly, CDK13 can phosphorylate the intracellular domain of the transmembrane protein SERINC5 within the cytoplasm (Chai et al., 2021). These observations indicate that CDK13 can be involved not just in transcription or RNA processing but also in other cellular processes in cytoplasm – although this needs further analyses.

### Deficiency in cell outgrowths as a potential cause of craniofacial anomalies

Our findings uncovered that *Cdk13*-deficiency triggers changes in the expression of neurogenesis-specific genes in the palatal shelves and the development of hypoplastic cranial nerves *in vivo*, confirming a previous *in-vitro* study using cortical neurons (Chen et al., 2014). However, a functional connection between anomalies in cranial nerve development, which, in turn, lead to facial malformations, has been detected only in few cases. Such non-canonical function of nerves as moderators of facial morphogenesis has been proven in Möbius syndrome, where either defective cranial nerves (Rizos et al., 1998; Tomas-Roca et al., 2015) or the entire defective rhombencephalon (Verzijl et al., 2003) can cause facial malformations. Other disorders of the peripheral nervous system that lead to facial anomalies are hereditary sensory and autonomic neuropathy type IV (HSAN IV) (Gao et al., 2013; Louryan et al., 1995; Nakajima et al., 2020) and Parry–Romberg syndrome with hemifacial atrophy accompanied by various neurological pathologies (Vix et al., 2015). Also, downregulated expression of the gene *Nrg1,* which was observed also by us, leads to altered maxillary development (Waddington et al., 2017), as this gene encodes neurogenin ligands with a positive axon growth potential. However, any functional association between the development of hypoplastic peripheral cranial nerves and the development of craniofacial clefts is still missing.

## Conclusions

CDK13 is crucial for the development of several tissues and organs, including craniofacial structures. Here, we determined its role during development of facial structures in a mouse model of CHDFIDD, where loss-of-function in *Cdk13* results in cleft lip/palate and the formation of midfacial clefts, accompanied by the deregulated expression pattern of certain genes and of proteins indispensable for proper functioning of major signaling pathways. *Cdk13*-deficient animals exhibited altered neurogenesis accompanied with distorted expression of neurogenesis-specific genes leading to the development of hypoplastic cranial nerves. As our analyses uncovered the cytoplasmic localization of CDK13 *in vitro*, further investigation is necessary to explore its potential roles beyond transcriptional regulation.

## MATERIALS AND METHODS

### Embryonic material

Heterozygous hypomorphic (*Cdk13^tm1a(EUCOM)Hmgu^*) and knockout (*Cdk13^tm1d^*) mice were obtained from the Infrafrontier Research Infrastructure – Mouse disease Models (https://www.infrafrontier.eu/services/rodent-model-generation/#MouseModelsGeneration). Mice were generated at the Transgenic and Archiving Module, CCP (IMG, Prague, Czech Republic).

Breeding and genotyping protocols were performed as previously described (see Nováková et al., 2019). In summary, the *Cdk13^tm1a^* allele consists of a splicing acceptor that is surrounded by two FRT sites within intron 2. Moreover, it has two *loxP* sites within intron 2 and one *loxP* site within intron 4. The *Cdk13^tm1d^* allele lacks exons 3 and 4, resulting in a non-functional allele of the *Cdk13* gene (Nováková et al., 2019).

All animal procedures were performed in strict accordance with the Guide for the Care and Use of Laboratory Animals and approved by the Institutional Animal Care and Use Committee (Masaryk University, Brno, Czechia, No. MSMT-34505/2020-7).

### Culture of MEF and NIH3T3 cells

Mouse embryonic fibroblasts (MEFs) were grown in conventional Dulbecco's modified Eagle medium (DMEM) supplemented with 4.5 g/l glucose and 20% fetal calf serum (FCS), the NIH3T3 cells were grown in DMEM supplemented with 4.5 g/l glucose and 10% FCS. Cell cultures were maintained at 37°C under 5% CO_2_.

### Live cell imaging

MEF cells isolated from WT and KO mouse embryos were cultured in DMEM as explained above. Cells were seeded on ibidiTreat μ-Slide 8 Well (80826, Ibidi) at concentration 50000/ml. After adhesion of cells, DMEM was changed to Opti-MEM (11058021, Gibco, Thermo Fisher Scientific) without Phenol Red supplemented with 1% fetal bovine serum (FBS). Cells were stained with F-actin-specific dye and membrane-specific dye 30 min before scanning. Live Imaging was performed using a Leica SP8 Confocal microscope (Leica, Germany) equipped with a CO_2_-controlled and tempered transparent chamber. Automated detection and tracking of filopodia, and automated quantification of cell migration was carried out as described by Barry et al. (2015). Final analysis was performed using GraphPad (GraphPad Software, Boston, MA, USA).

### Scanning electron microscopy

Mouse embryos (control and *Cdk13^tm1a/tm1a^*) were fixed in 4% paraformaldehyde, washed in distilled water and dehydrated through a graded series (30-100%) of ethanol solutions. Later, samples were dried out using the CPD 030 Critical Point Dryer (BAL-TEC) and shadowed by using gold in a metal shadowing apparatus Balzers SCD040 (Balzers, Liechtenstein). Images were taken with the TESCAN Vega TS 5136 XM scanning electron microscope (SEM) (Tescan, Czech Republic), using one embryo per stage (E12.5, E13.5, E14.5, E16.5) with the representative phenotype.

### Measurement of facial proportions

Frontal images of E11.5 embryonic heads were taken using a Leica S6D stereoscope with the DFC295 camera (both Leica, Germany). Individual measurements (mx – distance between edges of the maxillary prominences; lnp – distance between edges of the lateral nasal prominences; pits – distance between individual nasal pits) were performed in AxioVision 4.8 software (Zeiss, Germany) using length measurement tool. Distance ratios were calculated as *distance between nasal pits÷distance mx*, and *distance between nasal pits÷distance lnp*. Graphs and statistical significance were performed in GraphPad (GraphPad Software, Boston, MA, USA). Measurements were performed in at least four different embryos of all three genotypes (*Cdk13^+/+^*, *Cdk13^tm1a/tm1a^* and *Cdk13^tm1d/tm1d^*).

### Immunofluorescence on slides

Mouse embryonic tissues were fixed in 4% PFA overnight. Specimens were then embedded in paraffin and cut in transverse and sagittal planes at 5 µm sections. For immunohistochemistry staining, sections were deparaffinized in xylen and rehydrated in an ethanol series (100%, 96%, 70%). Antigen retrieval was performed either in 1% citrate buffer pH6 or in DAKO antigen retrieval solution pH9 (S1699, DAKO Agilent, USA) at 97.5°C.

For protein localization, we incubated sections with primary antibody (Table S5) for 1 h at room temperature or overnight at 4°C. Antibodies against the following proteins were used: 2H3 (1:50, Nefm, AB_2314897, Developmental Studies Hybridoma Bank), SOX2 (1:100, 2748s, Cell Signaling), SOX9 (1:100, HPA001758, Sigma), Ki67 (1:200, RBK027, Zytomed systems). Then sections were incubated with the following secondary antibodies (1:200) for 30 min at room temperature: anti-mouse Alexa-Fluor 488 (A11001), and anti-rabbit Alexa-Fluor 594 (A11037, both Thermo Fisher Scientific, USA). Ki-67-positive cells were detected by anti-rabbit secondary antibody, ABC binding (PK-6101, Vector laboratories) and followed by application of the DAB (K3468, Dako) chromogenic system.

For DNA staining (nuclei), sections were mounted using Fluoroshield with DAPI (F6057, Sigma, Merck, Germany). If DRAQ5 (62251, Thermo Fisher Scientific, USA) was used for nuclei staining, sections were mounted with Fluoroshield (F6182, Sigma, Merck, Germany). Pictures were taken on confocal microscopes Leica SP8 (Leica, Germany) and Zeiss LSM800 (Zeiss, Germany). Nuclei in DAB-stained sections were counterstained with hematoxilyn. Sections were photographed under bright-field illumination with the Leica DMLB2 compound microscope (Leica, Germany).

Mitotic index on Ki-67-stained sections in the palatal shelves, was counted as a ratio between Ki-67-positive cells (brown) and total number of cells (i.e. Ki-67-negative plus Ki-67-positive cells). Cells were counted independently in mesenchyme and epithelium of the palatal shelves. Cells were counted on four sections (both left and right palatal shelves) in three embryos for each genotype (*Cdk13^+/+^* and *Cdk13^tm1a/tm1a^*).

### Immunocytochemistry on glass inserts

MEFs and cells from embryonic DRGs (dorsal root ganglia) were isolated from E12.5 embryonic mice. Tissues were enzymatically processed using Dispase II (D4693, Sigma, Merck, Germany) for 1 h at 37°C while shaking. Cells were then centrifuged, filtered through 40 µm Cell strainer (431750, Corning), seeded on glass inserts and left to grow until 70–80% confluency in DMEM (D6546, Sigma, Merck, Germany). Adult DRG cells were isolated from DRGs from adult mice. DRGs were enzymatically processed using Collagenase IV (LS0004188, PAN Biotech) for 6 h at 37°C. Tissues were resuspended every hour by pipetting. Cells were then filtered through Cell strainer and seeded on glass inserts and left to grow and form long outgrowths in Neurobasal cultivation medium (21103-49, Gibco). Cells were then fixed in 4% PFA for 15 min.

For protein localization, we incubated cells on glass inserts with primary antibody for 1 h at room temperature. The following antibodies were used: anti-CDK13 (1:100, HPA059241, Sigma, Merck, Germany), anti-CDK13 N-TERM (1:100, SAB1302350, Sigma), anti-CDK13 (1:150, PA5-63692, Invitrogen, Thermo Fisher Scientific, USA), anti-F-actin (1:100, A12379, Alexa-Fluor 488™ phalloidin, Thermo Fisher Scientific), anti-2H3 (1:50, Nefm, AB_2314897, Developmental Studies Hybridoma Bank). Then sections were incubated with the following secondary antibodies (1:200) for 30 min at room temperature: anti-mouse Alexa-Fluor 488 (A11001), and anti-rabbit Alexa-Fluor 594 (A11037, both Thermo Fisher Scientific, USA). Glass inserts with cells were mounted on glass slides with Fluoroshield with DAPI (F6057, Sigma, Merck, Germany). Pictures were taken on confocal microscope Leica SP8 (Leica, Germany). Cells were cultivated from at least three different embryos of all three genotypes (*Cdk13^+/+^*, *Cdk13^tm1a/tm1a^* and *Cdk13^tm1d/tm1d^*).

### Whole-mount immunofluorescence

Mouse embryos were dissected, fixed in 4% PFA while rotating at 4°C for 4 h. Embryos were then postfixed in graded methanol dilutions (25%, 50%, 75%, 100%). Embryos were then bleached in a mixture of hydrogen peroxide, DMSO and methanol for 24 h, and postfixed in combination of DMSO and methanol. Embryos were incubated with primary antibody to stain neurofilaments (2H3, AB_2314897, Developmental Studies Hybridoma Bank) for 7 days while rotating, followed by secondary antibody incubation (anti-mouse Alexa-Fluor 488, A11001) for 2 days while rotating. Embryos were finally cleared in a mixture of benzyl benzoate and benzyl alcohol until they got transparent. For microscopy, embryos were placed on Nunc^TM^ glass bottom dishes (150680, Thermo Fisher Scientific) and imaged by using a Zeiss AxioZoom.V16-Apotome2 (Zeiss, Germany) at CELLIM (Core Facility Cellular Imaging, CEITEC, Masaryk University, Brno, Czech Republic). Whole-mount immunodetection of the neurofilaments was performed in three different embryos of all three genotypes (*Cdk13^+/+^*, *Cdk13^tm1a/tm1a^* and *Cdk13^tm1d/tm1d^*) at E11.5.

### Cellular fractionation

MEF and MIH3T3 cells were cultured on a 100 mm culture plate until 75% confluency in a 37°C incubator under 5% CO_2_. Cells were washed with ice-cold PBS and collected into 1 ml of ice-cold PBS with a scraper, transferred to an 1.5 ml Eppendorf tube and centrifuged at 300 ***g*** and 4°C for 4 min. Cell pellets were resuspended in 200 μl of TMK buffer (25 mM Tris-HCl pH 7.4; 1 mM MgCl_2_; 5 mM KCl) and 150 μl TMK+1% NP-40 was added to the tube. Incubation on ice was for 5 min, followed by centrifugation at 250 ***g*** and 4°C for 5 min. Supernatant (cytoplasmic fraction) was transferred to the new 1.5 ml Eppendorf tube. Pellet (nuclei) was resuspended in 500 μl of buffer S1 (0.25 M saccharose; 10 mM MgCl_2_) and transferred to the new Eppendorf tube on top of 500 μl of buffer S2 (0.35 M saccharose; 0.5 mM MgCl_2_), followed by centrifugation at 1400 ***g*** and 4°C for 5 min. Supernatant was removed and the pellet was resuspended in 50 μl of ice-cold PBS. Protein concentration was assessed for cytoplasmic and nuclear samples, and an appropriate volume of 3×Laemmli buffer was added to the given samples. The samples were boiled for 5 min at 100°C, followed by western blot procedure as published by Blazek et al. (2011). Antibodies used for western blotting were: CDK13 (Merck, cat. no.: HPA059241), PARP (Cell Signaling Technology, cat. no.: 9542S), α-Tubulin (Cell Signaling Technology, cat. no.: 7291S), Lamin B (Santa Cruz Technology, cat. no.: sc-6217) and GAPDH (Santa Cruz Technology, cat. no.: sc-23233).

### Chromatin immunoprecipitation (ChIP)

We employed the same chromatin immunoprecipitation protocol as published by Blazek et al. (2011), with few modifications: MEF cells were washed with PBS and crosslinked with 1% formaldehyde/PBS solution for 10 min at room temperature. Crosslinking was quenched for 5 min with 125 mM glycine (final concentration). Cells were washed twice with ice-cold PBS and lysed in sonication buffer (0.5% SDS, 20 mM Tris-HCl pH 8.0, 2 mM EDTA, 0.5 mM EGTA, 0.5 mM PMSF, protease inhibitor). For immunoprecipitation, protein extracts were pre-cleared with ChIP Grade G agarose beads (Cell Signaling Technology, cat. no.: 9007S) and then incubated overnight with anti-RNAPII antibody [Cell Signaling Technology, Rpb1 NTD (D8L4Y), cat. no.: 14958S; 1:300], followed by 2 h incubation with ChIP Grade G agarose beads. The beads were washed once with low-salt buffer (0.1% SDS, 1% Triton X-100, 2 mM EDTA, 20 mM Tris-HCl pH 8.0, 150 mM NaCl) followed by triple washing with the same buffer containing 500 mM NaCl, one wash with lithium buffer (2 mM EDTA, 20 mM Tris pH 8, 250 mM LiCl, 1% NP-40 and 1% natrium deoxycholate) and two washes with TE buffer. We eluted the immunoprecipitates with 1% SDS and 100 mM natrium bicarbonate at room temperature for 15 min and crosslinking was reversed by incubation with 200 mM NaCl for 5 h at 65°C. Proteins were digested with proteinase K (Sigma), and DNA was extracted with phenol:chloroform:isoamyl alcohol (25:24:1) and precipitated with isopropanol overnight. A fraction of precipitated DNA was used in qPCR reactions with the KAPA SYBR FAST qPCR Master Mix (2×) optimized for LightCycler^®^ 480 (Merck, KK4611). The amplifications were run using the Roche LC480II LightCycler under the following conditions: initial activation step at 94°C for 5 min, followed by 45 cycles at 95°C for 10 s, 60°C for 20 s and 72°C for 15 s. For the *Pou4f1* promoter, primers forward (Fw): 5′-AGAAATGCGCTGTGGATGAT-3′ and reverse (Re): 5′-TCCCGAGTAGAAAGCACACA-3′ were used as published by Tang et al. (2020). For the *Ntn1* promoter, primers Fw: 5′-GTCGGCAAATTTTCTCCAAA-3′ and Re: 5′-GTTGCATCCTTTCACCCACT-3′ were used as published by Kaneko et al. (2014).

### PCR array analysis to investigate gene-expression profiles

PCR arrays analyses were performed on tissues isolated from embryonic mice, i.e. rostral and caudal parts of palatal shelves at E12.5 and E14.5. One sample was obtained by pooling tissue from two or three embryos, three biological replicates were used for each stage and genotypes were analyzed. Total RNA was extracted using the RNeasy Plus Mini Kit (74136, Qiagen, Germany) according to the manufacturer′s instructions. Total RNA concentration and purity were measured using a NanoDrop One spectrophotometer (Thermo Fisher Scientific, USA). First-strand cDNA was synthesized using the gb Reverse Transcription Kit (3012, Generi Biotech, Czech Republic) according to the manufacturer′s instructions. RT² Profiler™ PCR Array Mouse Neurogenesis (330231, Qiagen, Germany) was performed according to the manufacturer′s instructions on a LightCycler 96 (Roche, Germany). C_T_ values were exported to an Excel file to create a table of C_T_ values. This table was then uploaded on to the data analysis web portal at http://www.qiagen.com/geneglobe. Samples were assigned to controls and test groups. C_T_ values were normalized to *Gapdh* as a reference gene. The above data analysis web portal calculated fold change/regulation using the delta delta C_T_ method, in which delta C_T_ is calculated between gene of interest (GOI) and an average of reference genes (HKG), followed by delta-delta C_T_ calculations [delta C_T_ (Test Group)-delta C_T_ (Control Group)]. Fold Change was then calculated using 2^ (-delta delta C_T_) formula. *P* values were calculated based on a Student's *t*-test of the replicate 2^(- Delta C_T_) values for each gene in control and test groups. The *P*-value calculation used is based on parametric, unpaired, two-sample equal variance, two-tailed distribution.

### RNAScope assay

Mouse embryos were fixed in 4% PFA and fixation time differed based on the stage. The tissues were then dehydrated in an ethanol series, embedded in paraffin, and 5 µm transverse sections were obtained. The sections were deparaffinized in xylene and dehydrated in 96% ethanol. To detect the expression of certain genes, we used RNA in situ hybridization assay (RNAScope Multiplex Fluorescent v2 Assay kit, 323 110, ACD Bio, USA) in formalin-fixed paraffin-embedded tissues according to the manufacturer's instructions. All reactions, which required incubation at 40°C, were carried out using the HybEZTM II oven (ACD Bio, USA). Probes for *Cdk13* (895581), *Fgf8* (313411), *FoxD1* (495501), *Meis2* (436371), *Msx1* (421841) and *Shh* (314361) (all ACD Bio, USA) were used. For negative control staining, a probe diluent (300041; ACD Bio) was used instead of a probe. The hybridized probes were visualized using the TSA-Plus Cyanine 3 system (NEL744001KT, Perkin-Elmer, USA), according to the manufacturer's protocol. DAPI (323 108, ACD Bio, USA) was used to stain nuclei. Pictures were obtained with the Leica SP8 confocal microscope (Leica, Germany).

RNAscope^®^ Probe Mm-*Cdk13* (#895581) binds to nucleotides 3312– 4317 (last couple of exons and the 3′UTR) of mouse *Cdk13* (NM_001081058.2). If deletion of exons 3 and 4 (*Cdk13^tm1d/tm1d^*) is unstable, it is possible to have a signal (some weak signal in the form of dots is present in both hypomorphic and knockout embryonic palatal shelves).

### RT-PCR

Rostral and caudal parts of the palatal shelves at E12.5, E14.5 and E16.5, as well as maxillary (mx), mandibular (md) and frontonasal prominences (fnp) at E11.5 and E12.5 were dissected from WT mouse embryos to quantify the differences in *Cdk13* expression during development of the facial structures. Individual parts of the palatal shelves and facial prominences were dissected from at least three different embryos for each stage. Proliferation rate changes in the *Cdk13*-deficient embryos were assessed using *CyclinD1* gene expression in tissues isolated from rostral and caudal palatal shelves (*Cdk13^tm1a^* E12.5, E14.5) and lips (*Cdk13^tm1d^* E12.5). Total RNA was extracted using the RNeasy Plus Mini Kit (74136, Qiagen, Germany) according to the manufacturer's instructions. Total RNA concentration and purity was measured using a NanoDrop One (Thermo Fisher Scientific, USA). First-strand cDNA was synthesized using gb Reverse Transcription Kit (3012, Generi Biotech, Czech Republic) according to the manufacturer's instructions. TaqMan probe was used to quantify *Cdk13* (Mm01164725_m1, Thermo Fisher Scientific) and *CyclinD1* (Mm00432359_m1, Thermo Fisher Scientific) gene expression. The RT-PCR reaction was performed on LightCycler 96 (Roche, Germany). The comparative C_T_ method was used for analysis.

### Injection of chicken embryos

Fertilized *Gallus gallus* (chicken) eggs were bought at a farm (Integra, Zabcice, Czech Republic) and incubated in a humidified incubator at 37°C. At Hamburger–Hamilton (HH) developmental stage 20 (HH20), 1 mM CDK12/13 inhibitor THZ531 (SML2619, Sigma Aldrich, Merck, Germany) was injected into the maxillary prominences (Fig. 8) using a glass capillary attached to an Eppendorf FemtoJet 4i Microinjector (Eppendorf, Germany) supplied with a manipulator (Leica, Germany). Moreover, we injected the inhibitor into the craniofacial mesenchyme and the postoptic region of chicken embryos at HH10 and HH15, respectively (Fig. 8). 10% Trypan Blue was used as a contrasting dye. After injections, embryos were incubated for another 96 h, and then killed and fixed in 4% paraformaldehyde. Frontal and lateral images were taken using a Leica S6D stereoscope with a DFC295 camera (both Leica, Germany).

### MTT-Assay

The MTT test was used to establish the toxicity of the CDK12/13 inhibitor THZ531. Cells isolated from the trigeminal ganglion (TG) were cultivated for 4 days in 100 μl of DMEM supplemented with 10% FBS, 1% penicilin/streptomycin, 1% L-glutamine in a 96-well plate (100,000 cells/ml). Cells were treated every day with different concentrations of THZ531 (i.e. 50, 100, 300, 500, 750, 1000 and 1500 nM). Untreated cells were used as controls. After 4 days, medium was discarded and replaced with 50 μl of DMEM without FBS followed by addition of 50 μl of MTT Reagent (MTT Cell Proliferation Kit Ab211091, Abcam), 50 μl of common culture medium and 50 μl of MTT Reagent were added to empty wells and used as background controls. The plate was then wrapped in foil and incubated for 3.5 h at 37°C under 5% CO_2_. Afterwards, 150 μl of MTT solvent (MTT Cell Proliferation Kit Ab211091, Abcam) was added to the samples. Absorbance was measured at 590 nm and cell survival rates were established as the absorbance index, with higher absorbance meaning better cell survival, lower absorbance meaning worse cell survival (Fig. S7H).

### TG cultivation and neurite outgrowth assay

TGs were dissected from mouse embryos at E12 and washed in ice-cold Neurobasal medium (21103049, Gibco, Thermo Fisher Scientific, USA). Each TG was placed in a separate well on a culture well plate into 10 μl drop of Matrigel (356231, Corning, USA) and left 20 min to polymerize in the tissue culture incubator. Neurobasal medium supplemented with 100 nM or 300 nM THZ531 (SML2619, Sigma Aldrich, Merck, Germany) was added. Images were taken after adding cultivation medium (0 h) and again after 24 h by using an Olympus IX71 inverted microscope (Olympus, Japan). Neurite outgrowth was measured using ImageJ software (NIH, USA) while comparing areas covered by neurites on pictures taken under different culture conditions.

Changes of the ability of TGs to produce neurite outgrowths (in %) were calculated as following: *neurite outgrowth area (total area−TG area)/total area×100*.

### Terminal deoxynucleotidyl transferase dUTP nick end labeling (TUNEL) assay

Apoptotic cells were detected by using the TUNEL assay (ApopTag Peroxidase In Situ Apoptosis Detection Kit, cat. no. S7101, Chemicon, Temecula, USA). Nuclei were counterstained with hematoxylin. Sections were photographed under bright-field illumination with a Leica DMLB2 compound microscope (Leica, Germany). TUNEL assays were performed using three different embryos of the *Cdk13^+/+^* and *Cdk13^tm1a/tm1a^* genotypes.

### Statistical analyses

Data were evaluated for statistical significance in GraphPad (GraphPad Software, Boston, MA, USA) using unpaired two-tailed Student's *t*-tests. Differences were considered to be significant at *P<0.05, **P<0.01 and **P<0.001. Errors are indicated as the ±standard error of the mean (±s.e.m.).

## Supplementary Material

10.1242/dmm.050261_sup1 Supplementary information

Table S1. E12.5 rostral Array excel report

Table S2. E12.5 caudal Array excel report

Table S3. E14.5 rostral Array excel report

Table S4. E14.5 caudal Array excel report
