# Supplementary material for: Early embryogenesis in CHDFIDD mouse model reveals facial clefts and altered cranial neurogenesis
Source: Dis Model Mech. 2024 Jun 20;17(6):dmm050261. doi: 10.1242/dmm.050261 (PMC11212636; doi:10.1242/dmm.050261)
Supplement: Supplementary information [file dmm-17-050261-s1.pdf]

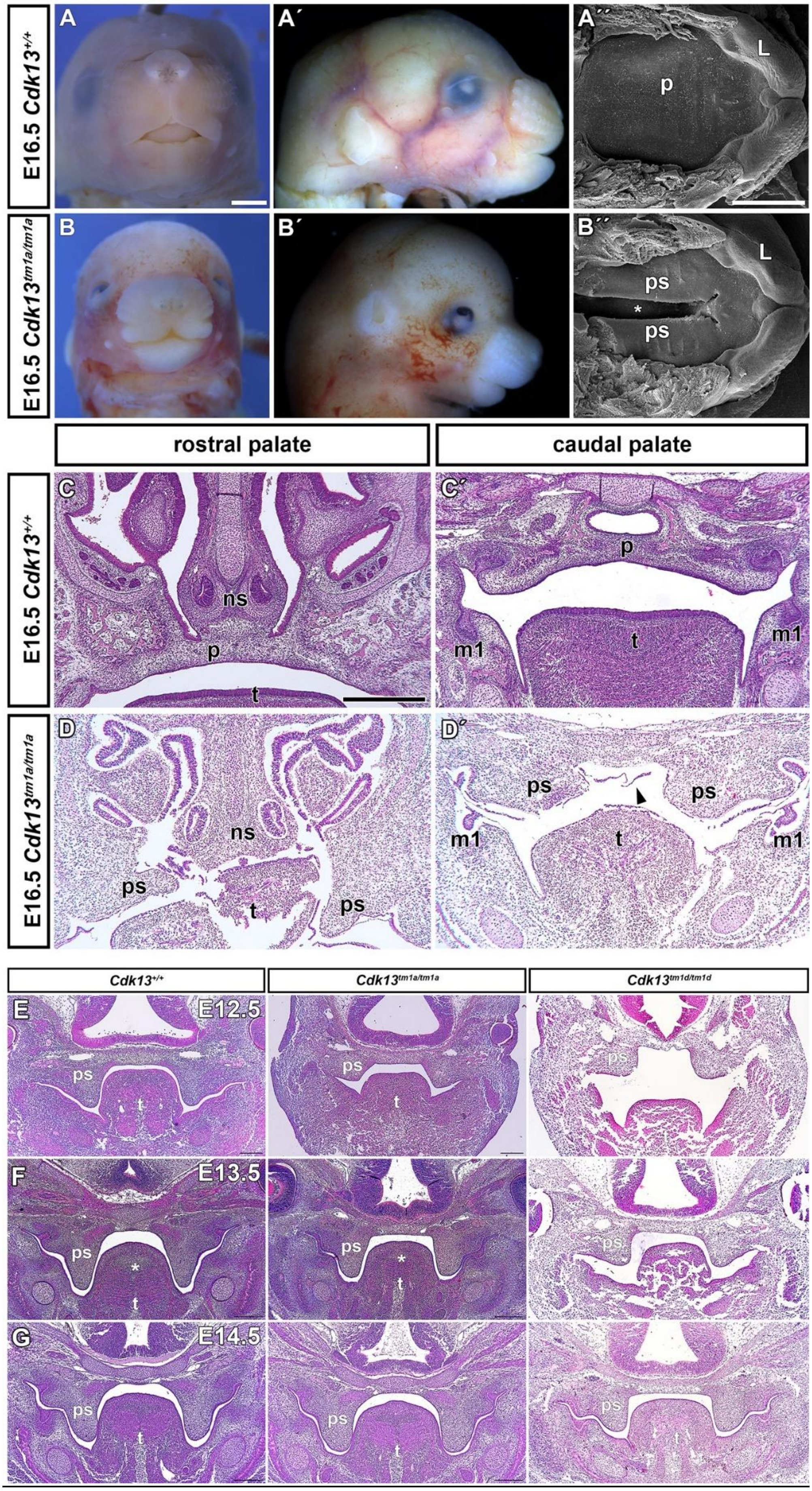

E16.5 Cdk13+/+
A
A´
A´´
L
p
E16.5 Cdk13tm1a/tm1a
B
B´
B´´
L
ps
*
ps
rostral palate
caudal palate
E16.5 Cdk13+/+
C
ns
p
t
C´
p
m1
t
m1
E16.5 Cdk13tm1a/tm1a
D
ns
ps
t
ps
D´
ps
ps
m1
t
m1
Cdk13+/+
Cdk13tm1a/tm1a
Cdk13tm1d/tm1d
E
E12.5
ps
t
ps
t
ps
F
E13.5
ps
*
t
ps
*
t
ps
G
E14.5
ps
t
ps
t
ps

Fig. S1. Craniofacial phenotype of *Cdk13*-deficient embryos.

(A-B´) External craniofacial phenotype of WT and *Cdk13*$^{tm1a/tm1a}$ embryos at E16.5 developmental stage in frontal and side views. Mutant embryos rather resemble earlier developmental stage. (**A´´, B´´)** Palatal view of the WT and mutant embryos using scanning electron microscope displaying the morphology of the palatal shelves, forming lips and vibrissae. Star marks visible cleft palate between palatal shelves in mutant embryo. (**C–D´)** Transversal Hematoxylin-Eosin-stained sections of the rostral and caudal palate in WT and mutant embryos at E16.5. Rostral area in *Cdk13*$^{tm1a/tm1a}$ embryo (**D**) is typical by the underdeveloped palatal shelves with intervening tongue between them resembling earlier developmental stage. In caudal palate (**D´´**, first molar level) of *Cdk13*$^{tm1a/tm1a}$ embryo, arrowhead points to visible cleft palate. L – lip, m1 – first molar, ns – nasal septum, p – palate, ps – palatal shelf, t – tongue. Scale bars: 1 mm.

(E-G) HE-stained transversal sections through the developing caudal palate at E12.5, E13.5 and E14.5 stages. Note underdeveloped palatal shelves (ps) in both mutant genotypes, close proximity of the neural tube with the oronasal cavity in E12.5 *Cdk13*$^{tm1d/tm1d}$ embryo and reduced tongue musculature in E13.5 mutant embryos (asterisk). ps – palatal shelf; t – tongue.

Disease Models & Mechanisms • Supplementary information

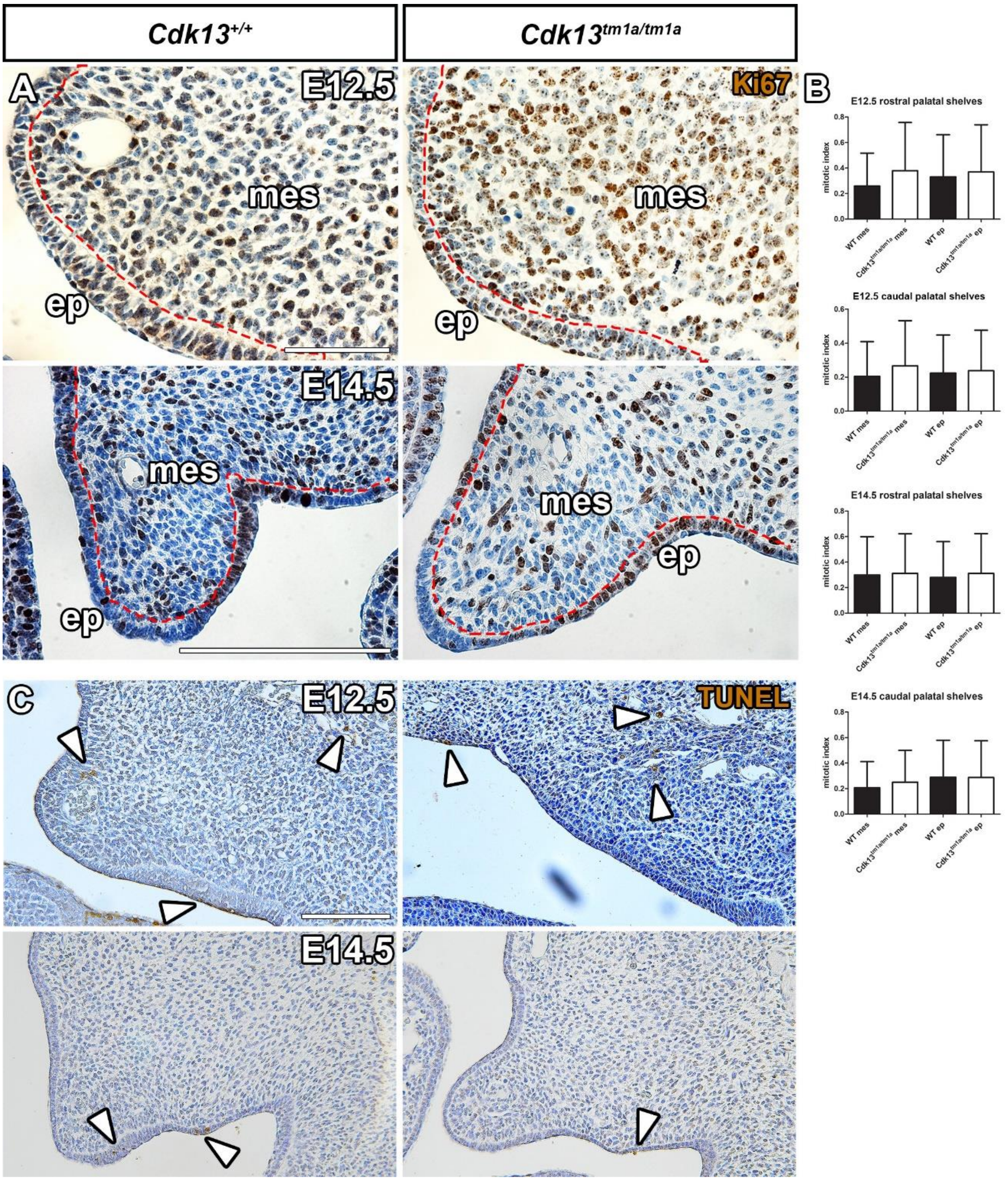

Fig. S2. Immunohistochemical detection of proliferating cells using Ki-67 antibody and detection of apoptotic cells by TUNEL Assay in the developing palatal shelves.

(A) Immunohistochemical detection of Ki-67-positive (brown) cells as a marker of proliferating cells in E12.5 and E14.5 palatal shelves on frontal sections. Palatal mesenchyme and epithelium are distinguished by a red dashed lines. Nuclei are counterstained with hematoxylin. Scale bars: 100 µm **(B)** Mitotic index counted from the Ki-67-positive cells detected by immunohistochemistry. Cells were counted independently in the rostral and caudal palatal shelves and separately in the palatal mesenchyme (mes) and epithelium (ep). Black columns represent WT embryos and white *Cdk13*$^{tm1a/tm1a}$ embryos. **(C)** Detection of the apoptotic cells by TUNEL assays on frontal sections of the E12.5 and E14.5 embryos. TUNEL-positivity is shown in brown, nuclei are counterstained by hematoxylin. Arrowheads point to positive signal. Scale bar: 500 µm. ep – epithelium; mes – mesenchyme.

Disease Models & Mechanisms • Supplementary information

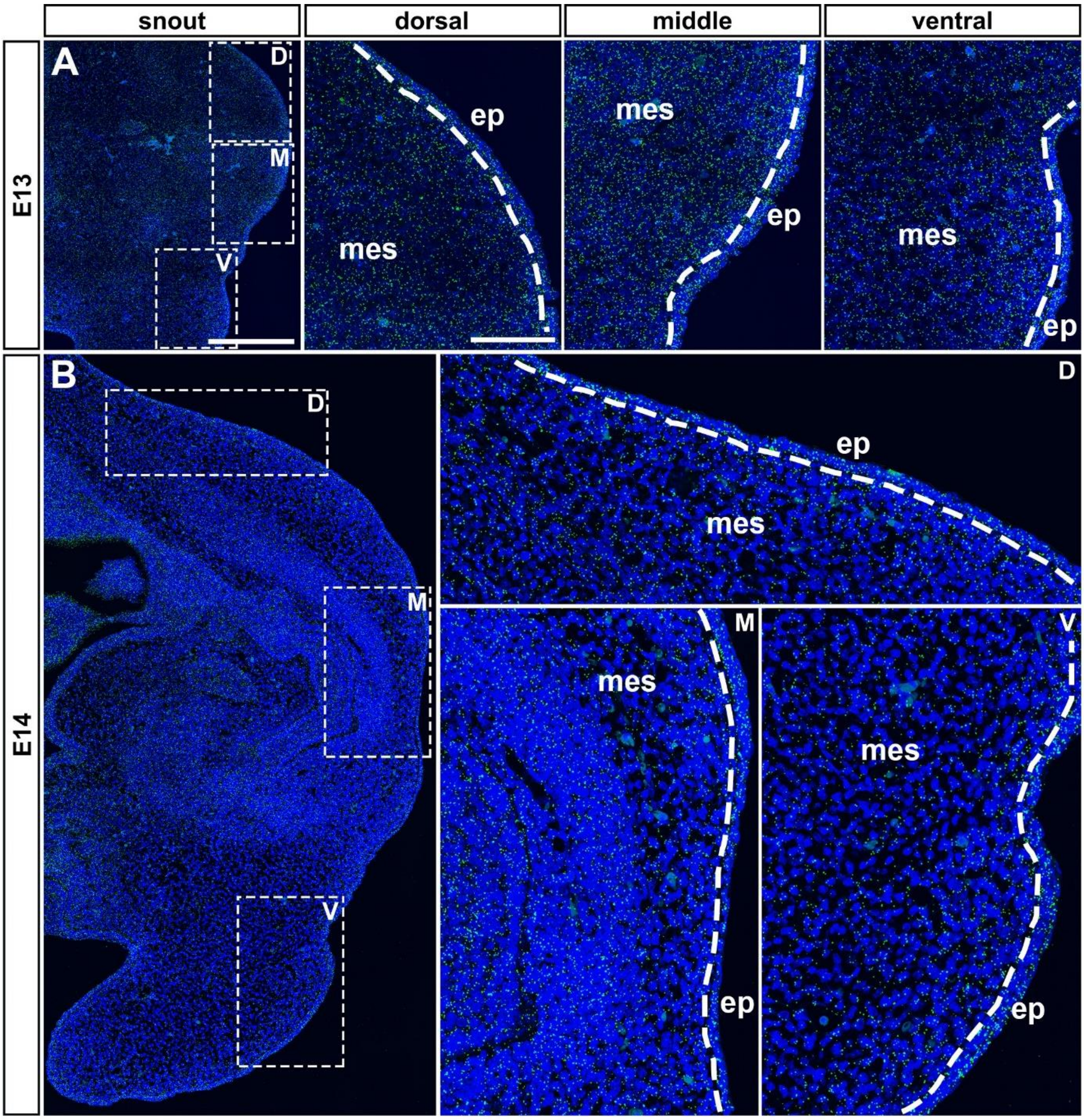

Fig. S3. Physiological expression of the *Cdk13* in the developing snout.

(A, B) RNAScope detection of the *Cdk13* (green) gene expression pattern in WT embryos. Lower power magnification of the *Cdk13* expression in the developing snout at E13 and E14 stages. White dashed line rectangles highlight regions used for higher power pictures in dorsal (D), middle (M) and ventral (V) areas. Scale bars: lower power – 300 µm; higher power - 100 µm. ep – epithelium; mes – mesenchyme.

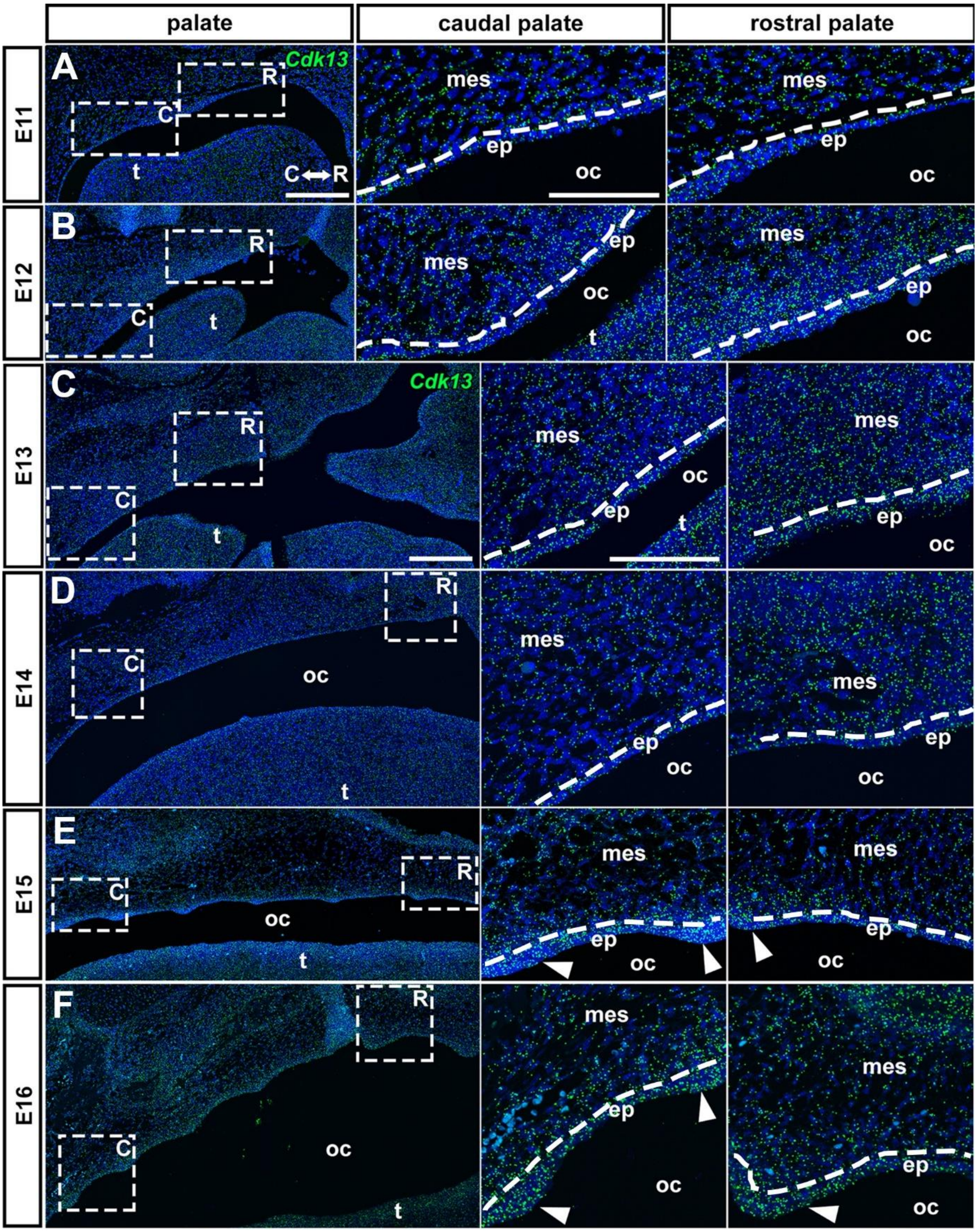

Fig. S4. Physiological expression of the *Cdk13* in the developing secondary palate.
(A–F) RNAScope detection of the *Cdk13* (green) in the WT palatal shelves from E12 to E16 stages sagittal sections. Lower power magnification of the *Cdk13* gene expression shows its distribution along rostral-caudal axis. White dashed line rectangles highlight regions used for higher power pictures in caudal **(C)** and rostral **(**R**)** regions. Arrowheads point to dense expression of the *Cdk13* in the palatal ridges in E15 and E16 embryos. Scale bars: lower power – 200 µm; higher power – 100 µm. ep – epithelium; mes – mesenchyme; oc – oral cavity; t – tongue.

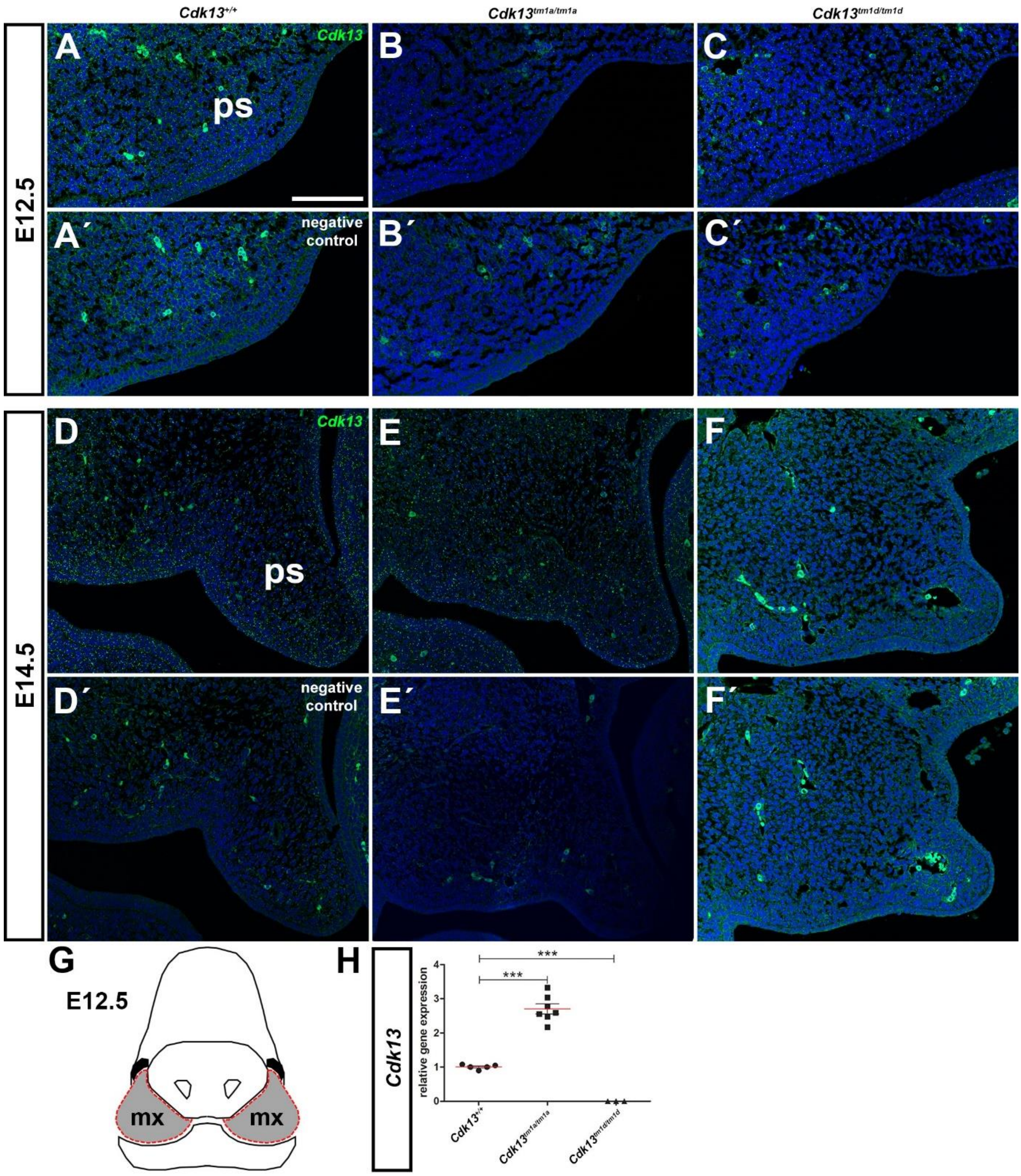

Fig. S5. *Cdk13* gene expression in the facial structures.

(A, A´) RNAScope detection showing pictures with the *Cdk13* (green) gene expression and negative control staining in the forming palatal shelf of E12.5 WT embryo, **(B, B´)** E12.5 *Cdk13*$^{tm1a/tm1a}$ and in **(C,C´)** E12.5 *Cdk13*$^{tm1d/tm1d}$ embryos. **(D,D´)** Pictures showing *Cdk13* (green) gene expression and negative control staining in the forming palatal shelf of E14.5 WT embryo, **(E, E´)** E14.5 *Cdk13*$^{tm1a/tm1a}$ and in **(F,F´)** E14.5 *Cdk13*$^{tm1d/tm1d}$ embryos. Note the specific RNAScope expression pattern shown as dots present in *Cdk13* stained sections and missing in negative control samples. Scale bar: 100 µm. **(G)** Schematic description what tissues and how were dissected for quantification of the *Cdk13* gene expression. **(H)** Quantification of the *Cdk13* expression using qPCR in E12.5 maxillary prominences of all the three genotypes. The used TaqMan probe spans exons 3 and 4 (exons missing in the *Cdk13*$^{tm1d/tm1d}$ animals). Unpaired two-tailed Student t - test; **$0.001 < p < 0.01$; *$p < 0.05$.

Disease Models & Mechanisms • Supplementary information

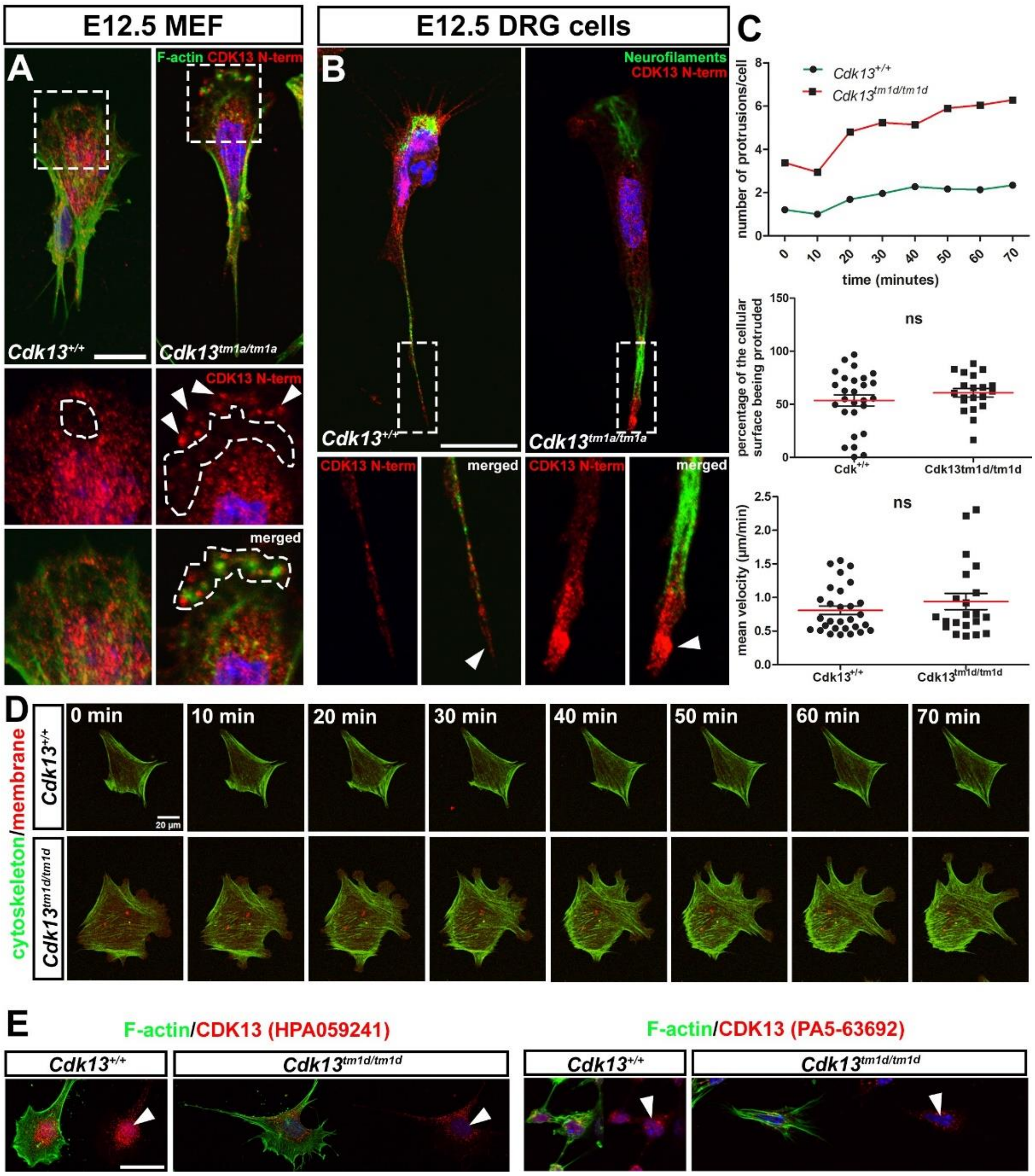

Fig. S6. Immunocytochemical localization of the CDK13 in control and *Cdk13*-deficient cells.
(A) Detection of the truncated form of CDK13 (red) in E12.5 *Cdk13*$^{tm1a/tm1a}$ MEF cells using anti N-terminal end CDK13 antibody. Dashed line rectangles highlight details on cellular outgrowths. Detailed pictures focus on aggregates of the truncated CDK13 in the cellular protrusions (arrowheads), CDK13-negative area (dashed line region, red) and colocalization of the truncated CDK13 with F-actin (green) deposits (dashed line region, merged). **(B)** Detection of the truncated form of CDK13 (red) in E12.5 *Cdk13*$^{tm1a/tm1a}$ DRG cells using anti N-terminal end CDK13 antibody colocalized with neurofilaments (green, anti-2H3). Dashed line rectangles highlight details on long cellular outgrowths. Detailed pictures focus on accumulation of the truncated CDK13 in distal part of the cellular outgrowth (arrowhead, merged). **(C)** Graphical representation of cells being tracked using Live Cell Imaging. Upper graph displays several protrusions per cell produced during time. Middle dot plot demonstrates percentage of the cellular surface protruded from cells. Lower dot plot displays comparison of the control and KO cells in the ability to move (mean velocity, micrometers per minute). **(D)** Changes of the morphology of control and KO cells in time using Live Cell Imaging. Green color represents cytoskeleton, red color highlights membrane. These cells were picked as cells with representative phenotype specific for each genotype. **(E)** Left panel shows protein expression of the CDK13 in control and KO cells using HPA059241 antibody. Right panel displays protein expression of the CDK13 in control and KO cells using PA5-63692 antibody. Nuclei were counterstained with DAPI. Arrowheads point to signal detected using anti-CDK13 antibodies (red) in nuclei. Scale bars: (A) – 10 µm; (B) – 20 µm; (D,E) – 20 µm.

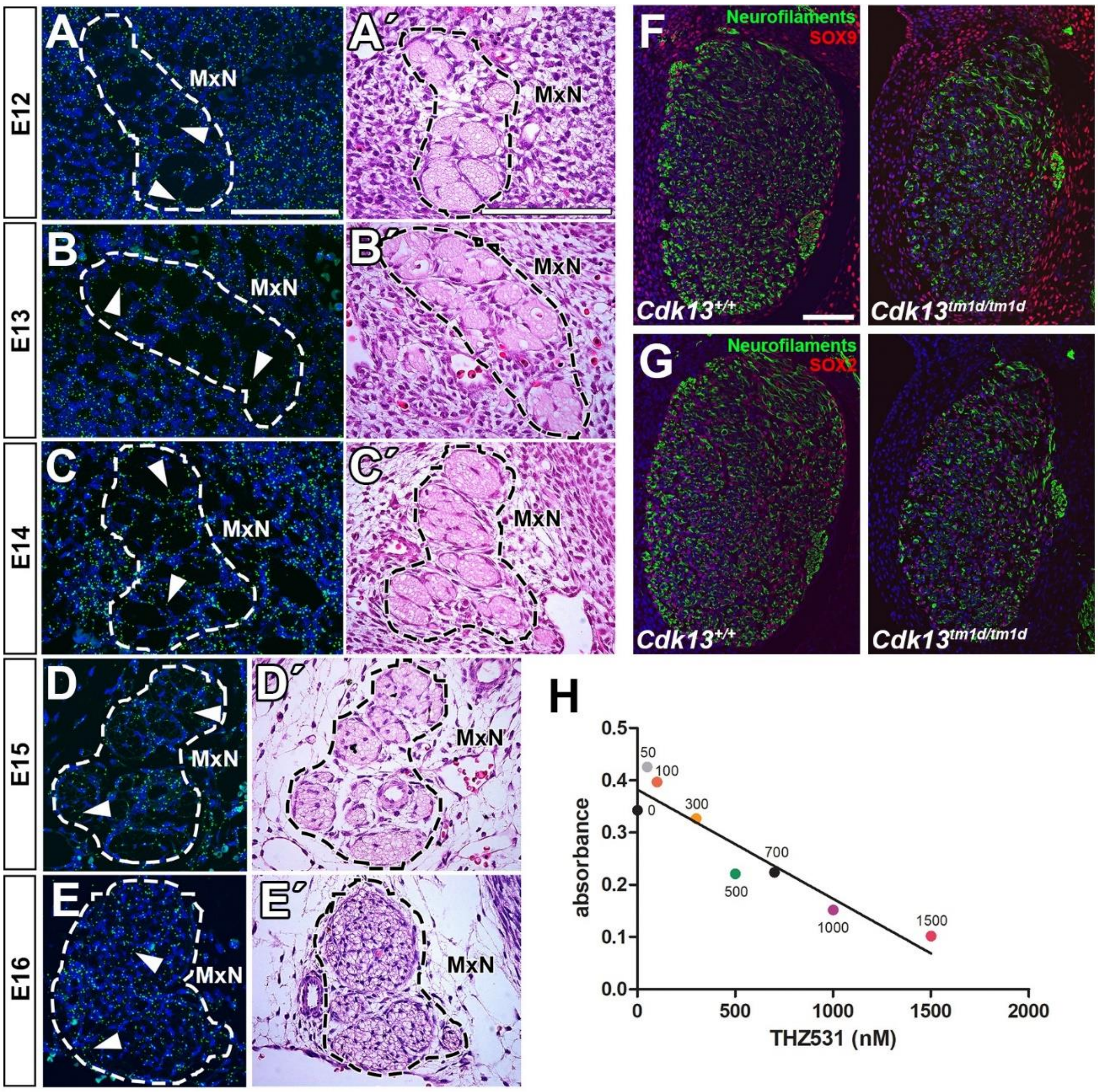

Fig. S7. *Cdk13* expression and the effect of its downregulation on development of trigeminal ganglion.

RNAScope detection of the *Cdk13* in maxillary nerve.

(A-E) RNAScope detection of the *Cdk13* expression in the forming maxillary nerve. Arrowheads point to *Cdk13* localization in the cellular outgrowths protruding to the developmental nerves. Dashed line areas outline nerves. Nuclei are counterstained with DAPI. **(A´-E´)** HE-stained alternative sections display formation of the maxillary nerve. Scale bar: µm 100. MxN – maxillary nerve.

Immunohistochemical detection of neurofilaments and SOX-family proteins in trigeminal ganglion.

(F,G) Immunohistochemical detection of neurofilaments (green), SOX9 (red) and SOX2 (red) in the developing trigeminal ganglion in E11.5 embryos on transversal sections. Note reduced size of the trigeminal ganglion in $Cdk13^{tm1d/tm1d}$ embryo. Nuclei are counterstained with DAPI. Scale bar: 100 µm.

THZ531 cytotoxicity tested by MTT Assay.

(H) A graph showing decreasing absorbance with the increasing cytotoxicity of the THZ531 inhibitor used on cells isolated from the trigeminal ganglions. 100 nM and 300 nM concentrations were than used to treat trigeminal ganglia in ex vivo cultivation experiments.

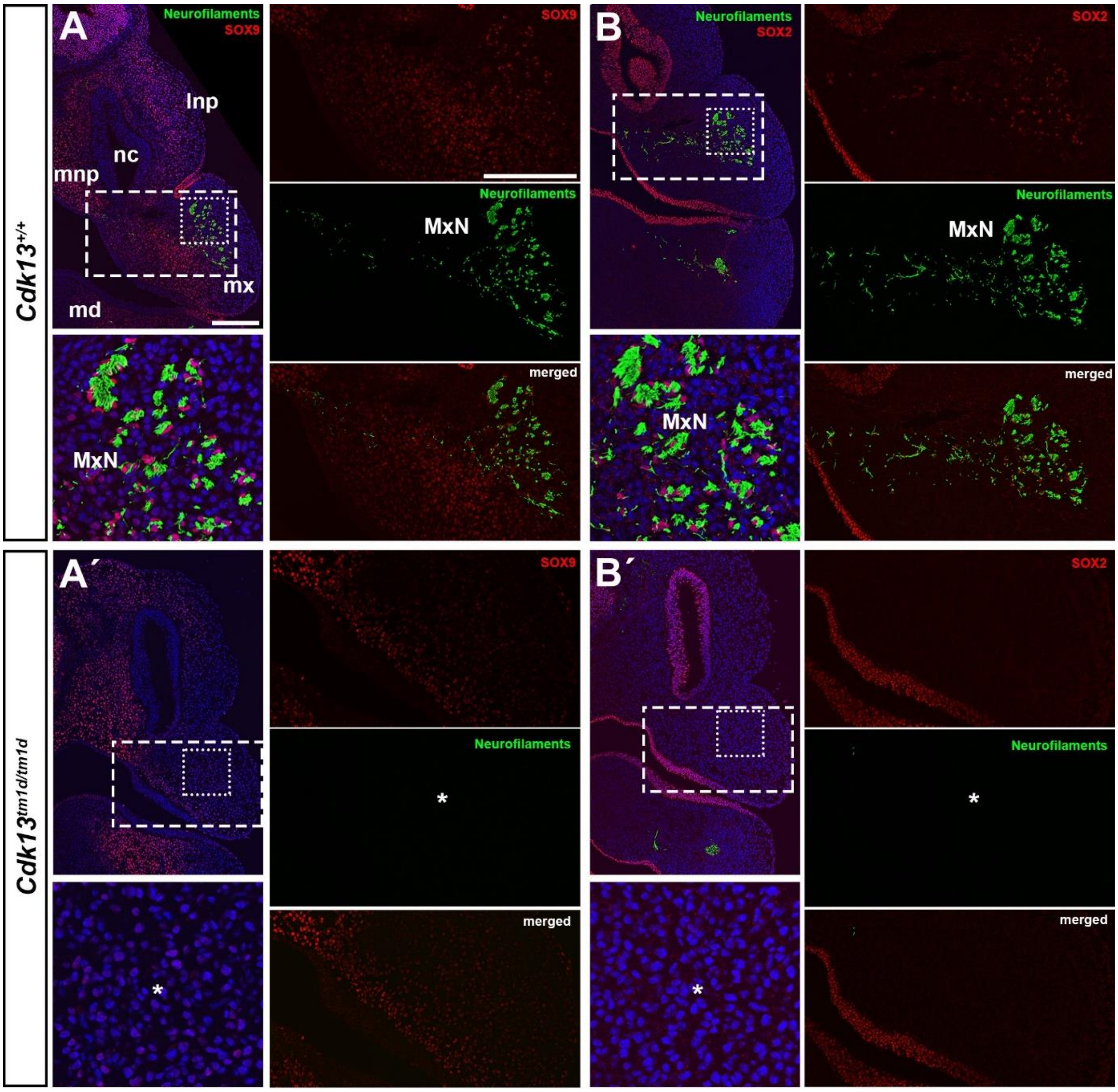

Fig. S8. Immunohistochemical detection of neurofilaments, SOX9 and SOX2 on frontal sections of E11.5 embryos.

(A-B´) Lower power pictures depicting IHC detection of neurofilaments (2H3-antibody) co-stained with SOX9 or SOX2 antibody in the facial prominences at E11.5. White dashed line rectangles highlight individual pictures showing split channels (SOX9 or SOX2 – red; neurofilaments – green) and merged channels (white). White dotted line squares highlight individual higher power pictures with details of maxillary nerves (green) with cells expressing either SOX9 or SOX2 (red). White asterisks focus to the area with missing maxillary branch of the TG nerve. Scale bars: lower power - 200 µm; higher power - 200 µm. lnp– lateral nasal prominence; mnp – medial nasal prominence; md– mandibular prominence; mx– maxillary prominence; MxN – maxillary branch of the trigeminal nerve; nc – nasal cavity.

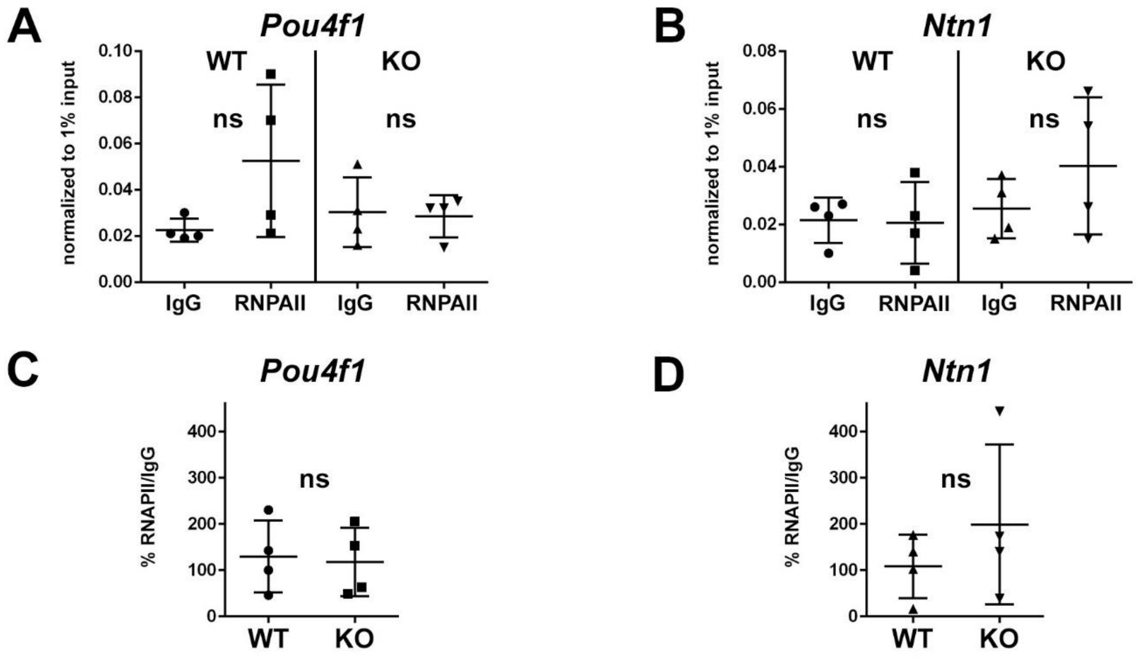

Fig. S9. Depletion of CDK13 leads to lower and higher occupancy of RNAPII within promoters of *Pou4f1* and *Ntn1* genes.

(A-B) ChIP analyses for the occupation of RNAPII on the promoters of indicated genes. IgG corresponds to the empty beads control. Experiments are the results of three independent experiments, and qPCR was performed in duplicate. No statistical significance of biological replicates was confirmed at $P < 0.05$. **(C-D)** Percentage of RNAPII binding to promoter sequence was calculated to IgG signal.

Disease Models & Mechanisms • Supplementary information

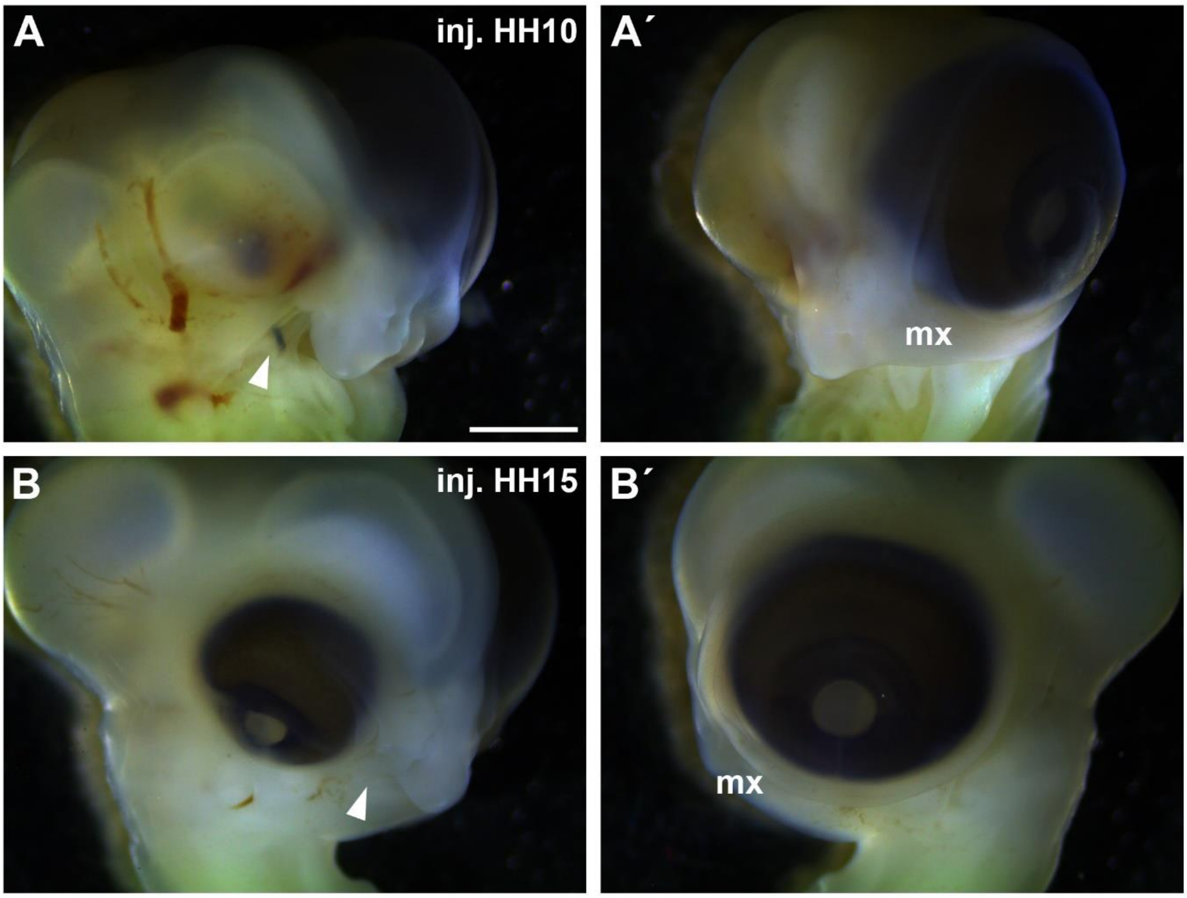

C

injection stage	phenotype frequency
HH10	100 % (6/6)
HH15	86 % (6/7)
HH20	83 % (20/24)

Fig. S10. Chemical inhibition of the CDK12 and CDK13 leads to embryonic facial malformations in chicken model.

(A-B´) Macroscopic pictures focused on lateral regions of the embryonic head. **(A,B)** Missing right maxillary prominences visible in both **(A)** chickens injected at HH10 and **(B)** at HH15 highlighted by white arrowheads. **(A´,B´)** Left control untreated (not injected) sides with no defects. Scale bar: 2 mm. mx – maxillary prominence. **(C)** Table displaying number of chickens with phenotype based on injection stage with THZ531 inhibitor.

Disease Models & Mechanisms • Supplementary information

Table S1. E12.5 rostral Array excel report

Available for download at
https://journals.biologists.com/dmm/article-lookup/doi/10.1242/dmm.050261#supplementary-data

Table S2. E12.5 caudal Array excel report

Available for download at
https://journals.biologists.com/dmm/article-lookup/doi/10.1242/dmm.050261#supplementary-data

Table S3. E14.5 rostral Array excel report

Available for download at
https://journals.biologists.com/dmm/article-lookup/doi/10.1242/dmm.050261#supplementary-data

Table S4. E14.5 caudal Array excel report

Available for download at
https://journals.biologists.com/dmm/article-lookup/doi/10.1242/dmm.050261#supplementary-data

Disease Models & Mechanisms • Supplementary information

Table S5. List of used primary antibodies

Antibody	Retrieval	Dilution	Cat.No.	Company	Notes
2H3	DAKO, pH 9 (IHC)	1:50	AB_2314897	Developmental Studies Hybridoma Bank	Mouse
SOX2	DAKO, pH 9 (IHC)	1:100	2748s	Cell Signaling	Rabbit
SOX9	DAKO, pH 9 (IHC)	1:100	HPA001758	Sigma	Rabbit
Ki67	1% Citrate buffer, pH 6 (IHC)	1:200	RBK027	Zytomed Systems	Rabbit
CDK13	N/A (ICC)	1:100	HPA059241	Sigma	Rabbit
CDK13 N-term	N/A (ICC)	1:100	SAB1302350	Sigma	Rabbit
CDK13	NA (ICC)	1:150	PA5-63692	Invitrogen	Rabbit
F-Actin	N/A (ICC)	1:100	A12379	Thermo Fisher	
2H3	N/A (ICC, Whole Mount IHC)	1:50	AB_2314897	Developmental Studies Hybridoma Bank	Mouse
CDK13	N/A (WB – cell fractionation)	1:3000	HPA059241	Sigma	Rabbit
PARP	N/A (WB – cell fractionation)	1:3000	9542S	Cell Signaling Technology	Rabbit
α-Tubulin	N/A (WB – cell fractionation)	1:10000	7291S	Cell Signaling Technology	Rabbit
Lamin B	N/A (WB – cell fractionation)	1:1000	sc-6217	Santa Cruz Technology	Goat
GAPDH	N/A (WB – cell fractionation)	1:3000	sc-32233	Santa Cruz Technology	Mouse
RNAPII	N/A (ChIP)	1:300	14958S	Cell Signaling Technology	Rabbit

Disease Models & Mechanisms • Supplementary information
